# Biomechanical and biochemical assessment of YB-1 expression in A375 melanoma cell line: Exploratory study

**DOI:** 10.3389/fmmed.2023.1050487

**Published:** 2023-04-20

**Authors:** Anna Cykowska, Ulf Krister Hofmann, Aadhya Tiwari, Corinna Kosnopfel, Rosa Riester, Marina Danalache

**Affiliations:** ^1^ Department of Orthopaedic Surgery, University Hospital and Faculty of Medicine, University Hospital of Tübingen, Tübingen, Germany; ^2^ Department of Clinical and Biological Sciences, University of Turin, Orbassano, Italy; ^3^ Department of Orthopedic, Trauma and Reconstructive Surgery, RWTH Aachen University Hospital, Aachen, Germany; ^4^ Department of System Biology, MD Anderson Cancer Center, Houston, TX, United States; ^5^ Department of Dermatology, Venereology and Allergology, University Hospital Würzburg, Würzburg, Germany; ^6^ Department of Hematology, Oncology, and Pneumology, University Hospital Münster, Münster, Germany

**Keywords:** melanoma, atomic force microscopy, YB-1, omics, proteomics, cytoskeleton, migration, melanoma

## Abstract

Malignant melanoma is the most lethal form of skin cancer. Y-box binding protein 1 (YB-1) plays a prominent role in mediating metastatic behavior by promoting epithelial-to-mesenchymal transition (EMT). Migratory melanoma cells exhibit two major migration modes: elongated mesenchymal or rounded amoeboid. Using A375 melanoma cell line and the YB-1 knock-out model, we aimed to elucidate biochemical and biomechanical changes in migration signaling pathways in the context of melanoma metastases. We subjected A375 YB-1 knock-out and parental cells to atomic force microscopy (stiffness determination), immunolabelling, and proteome analysis. We found that YB-1 expressing cells were significantly stiffer compared to the corresponding YB-1 knock-out cell line. Our study demonstrated that the constitutive expression of YB-1 in A375 melanoma cell line appears to be closely related to known biomarkers of epithelial-to-mesenchymal transition, nestin, and vimentin, resulting in a stiffer phenotype, as well as a wide array of proteins involved in RNA, ribosomes, and spliceosomes. YB-1 knock-out resulted in nestin depletion and significantly lower vimentin expression, as well as global upregulation of proteins related to the cytoskeleton and migration. YB-1 knock-out cells demonstrated both morphological features and biochemical drivers of mesenchymal/ameboid migration. Melanoma is a highly plastic, adaptable, and aggressive tumor entity, capable of exhibiting characteristics of different migratory modes.

## 1 Introduction

Malignant melanoma is the most lethal form of skin cancer. Melanoma is a particularly aggressive cancer and the prognosis for affected patients is grim with an estimated 5-year survival rate ranging between 5%–19% (Sandru et al., 2014). At the cellular level, melanoma utilizes a combination of several mechanisms to invade tissues: epithelial-mesenchymal transition (EMT), loss of cell-to-cell adhesion, a loss of cell-matrix adhesion, matrix degradation, and mesenchymal/ameboid migration (Son and Moon, 2010). Y-box binding protein 1 (YB-1) is a multifunctional member of the cold-shock protein superfamily that has a dual role as a transcription factor in the nucleus and a translational regulator in the cytoplasm (Lasham et al., 2013; Kosnopfel et al., 2014). YB-1 expression predicts drug resistance and patient outcome in many tumors and is often linked to patient relapse and poor prognosis in a plethora of cancer types (Habibi et al., 2008; Shiota et al., 2008; Basaki et al., 2010). Furthermore, in melanoma, YB-1 mediates metastatic behavior (Lu et al., 2017) and is a biomarker of exacerbated tumor progression (Ferreira et al., 2017). Kosnopfel et al. showed that YB-1 triggers tumorigenicity and metastatic potential of melanoma cells by promoting epithelial-to-mesenchymal transition (EMT)-like processes (Kosnopfel et al., 2018). The impact of YB-1 impact on migration signaling pathways in the context of melanoma metastases is poorly understood. When melanoma cells detach from the primary tumor site, they can move in several ways. The two main modes are the “path generating” mesenchymal and the “pathfinding” amoeboid, which are up to 20 times more invasive in some cancers than the mesenchymal phenotype (Vig et al., 2017). Mesenchymal cells use proteolysis to degrade the surrounding extracellular matrix and create new paths for them to move through, whereas amoeboid cells are more flexible and can squeeze through small gaps to find their way, but they cannot degrade the ECM or create new paths (Yu et al., 2013; Hecht et al., 2015). Mesenchymal migration is a protrusion‐dependent mode and is characterized by focal adhesion formation and lower actomyosin contractility (Sanz-Moreno et al., 2008). Rounded amoeboid invasion uses unstable blebs that can squeeze through the matrix, therefore exhibiting a lower degree of β1integrin‐mediated adhesion, reduced focal adhesion size, high actomyosin contractility, blebs formation, and high cytokine signaling (Charras and Paluch, 2008). From a structural perspective, the cytoskeleton comprises three main components: the actin cytoskeleton, the microtubule network, and the intermediate filaments (IF). Although these components act synergistically, the driving force required for cell migration is the actin cytoskeleton (Chan et al., 2014). Alongside the actin cytoskeleton, microtubules also regulate metastatic potential *via* crosstalk with actin (Eitaki et al., 2012). Coexisting with actin and microtubules, intermediate filaments are responsible for traction forces between cells and protect the cells from disruptions. Throughout EMT, intermediate filaments undergo a significant rearrangement process that switches from cytokeratin-rich to vimentin- and nestin-rich networks, resulting in increased cell motility capacity (Sun et al., 2015). Melanoma has been previously shown to be able to utilize both modes of invasion, however, the biochemical and biomechanical signaling cascade driving those changes remain unclear. There is a need to identify potential biomarkers both pathways, allowing to predict amoeboid invasion and metastatic progression and it is highly relevant to know which proteins are involved in the switch from ameboid to mesenchymal phenotype. To elucidate such biochemical and biomechanical changes induced by YB-1 expression we used A375 melanoma cell lines including YB1 expressing melanoma cell line, and A375 YB1 knockdown cells. Not only do we aim to investigate to what extent YB-1 impacts the biochemical properties of melanoma cells and their cytoskeleton, but we also aim to investigate the adaptability of melanoma in the absence of YB-1. We also assessed the cell stiffness of both cell lines in relation to biochemical changes as a potential prognostic biomarker of invasiveness.

## 2 Materials and methods

### 2.1 Cell lines and culture

A375 and MelJuso cells (ATCC, Manassas, VA, United States and DSMZ, Braunschweig, Germany) were used in this study, parental (not modified) and knock-out (described in section 2.2).

Both cell lines were maintained in RPMI-1640 with L-glutamine (Gibco, Life Technologies, Darmstadt, Germany) supplemented with 10% (v/v) fetal calf serum (Merck Biochrom, Berlin, Germany) and 1% (v/v) penicillin/streptomycin (Thermo Fisher Scientific, Waltham, MA, United States) in an incubator at 37°C as previously described (Kosnopfel et al., 2020). For all experiments, cells with passage numbers of 5–20 at approximately 80% confluency were harvested. All melanoma cell lines were used for no longer than 2 months after thawing from the frozen stock.

### 2.2 YB-1 knock-out in A375 cells

A YB-1 gene knockout (CRISPR YB-1^KO^) for an A375 cell line using CRISPR/Cas9-mediated genome engineering and single-cell clones with effective YB-1 knock-out were identified as previously described (Kosnopfel et al., 2018; Kosnopfel et al., 2020). The sequences of the sgRNAs used for YB-1 knock-out along with the lentiviral transfer vector (lentiCRISPRv2) used for melanoma transduction were also previously described (Kosnopfel et al., 2017). Successful knockout of YB-1 in A375 cell line was previously confirmed by lack of protein expression in Western blot analysis (Kosnopfel et al., 2018).

### 2.3 Biomechanical characterization by atomic force microscopy (AFM)

**FIGURE 1:**
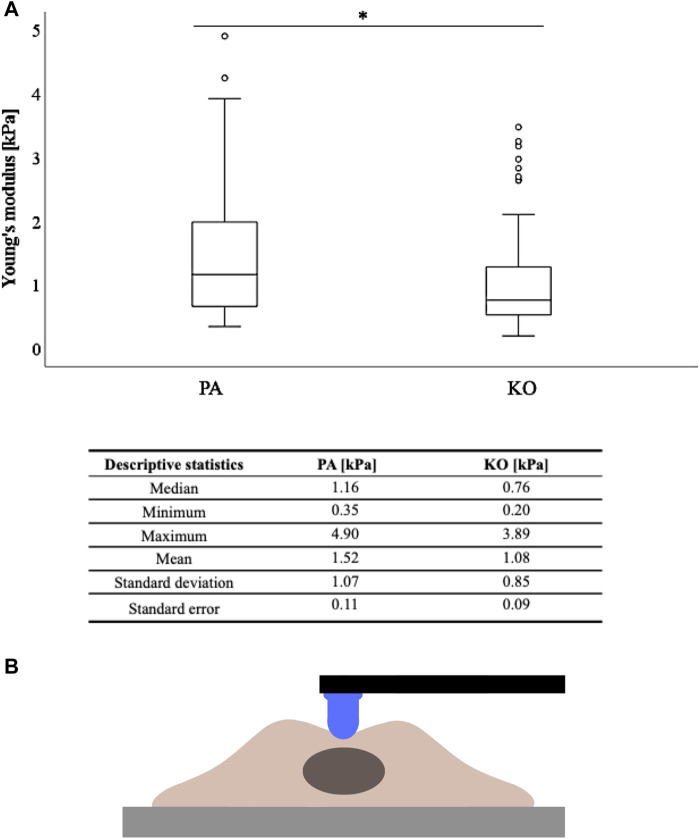
Microscopic images representing the regions of interest subjected to AFM measurements. **(A)** Cell lines A375 parental and A375 YB-1 knock-out, as well as MelJuso parental (Supplementary Figure S1), were subjected to AFM measurements. White arrows indicate the cantilever employed for indentation. Black arrows show the region of interest to be measured - an exemplary cell. **(B)** Schematic drawing of a single cell indented by a spherical AFM cantilever (5 µm). Images were acquired with the inverted AxioObserver D1 light microscope attached to the AFM system at × 10 magnification. The scale bar (black) represents 30 µm. Abbreviations: AFM - atomic force microscopy, PA - parental, KO - knock-out.

To measure the stiffness of cells, we used an atomic force microscope (AFM) system (CellHesion 200, JPK Instruments, Berlin, Germany) mounted onto an inverted light microscope (AxioObserver D1, Carl Zeiss Microscopy, Jena, Germany), which allowed us to optically align the cantilever to the region of interest and to simultaneously measure specific regions of interest on individual cells (Figure 1). The calibration of the cantilever was done on an empty Petri dish filled with pre-warmed to 37°C Leibovitz’s L-15 medium without L-glutamine (Merck KGaA, Darmstadt, Germany). The calibration was performed on the retracted curve and the spring constant was determined by using the thermal noise method incorporated into the device software (JPK Instruments, Berlin, Germany). Measurements were made in force spectroscopy mode by recording single force-distance curves at the position of interest without laterally scanning the sample. Indentation was performed using a spherical tip of 5 µm (Model: SAA-SPH-5UM, k = 0.2 N/m, Bruker, Billerica, MA, United States). Cells were grown for 48 h prior to the AFM biomechanical measurements on the bottom of the dishes (TPP Techno Plastic Products AG, Trasadingen, Switzerland) until sufficient confluency was reached (±80%). The culture medium (described in 2.1) was changed 24 and 48 h after seeding. Prior to mechanical measurements, the culture medium was removed, and the cells were covered with Leibovitz’s L-15 medium without L-glutamine (Merck KGaA). To eliminate the confounding effects of neighboring cells on cytoskeleton arrangement and morphology, randomly selected single cells were measured (Figure 1). To evaluate the elastic properties of A375 and MelJuso cells, we indented the selected cells approximately over the perinuclear region of individual cells. Indentation curves were sampled at 2 kHz, with a force trigger of ∼10 nN and a velocity of 5 μm/sec. We measured 30 cells per condition and each cell was automatically measured five times at a single measurement side. Overall, we measured all cell lines in three independent repetitions. The Young’s modulus for each individual cell was calculated from the force-distance curves from each measurement by using the Hertz fit model incorporated in the data processing software (JPK Instruments), with Poisson’s ratio set to 0.5. Alongside A375 cells, MelJuso YB-1 expressing cells were also subjected to cellular stiffness measurements to investigate the possible heterogeneity of elastic features that may vary within cancerous tumors.

### 2.4 NanoLC-MS/MS analysis and data processing

Prior to the proteome analysis, YB-1 presence/absence in cells lines was verified *via* Western blot analysis as previously described (Kosnopfel et al., 2018). For the proteomic investigations, protein extracts were purified using SDS PAGE (Thermo Scientific). Coomassie-stained gel pieces were excised and in-gel digested using trypsin as described previously (Borchert et al., 2010). After desalting using C18 Stage tips, extracted peptides were separated on an Easy-nLC 1200 system coupled to a Q Exactive HF mass spectrometer (both Thermo Scientific) as described elsewhere by Kliza et al. (Kliza et al., 2017) with slight modifications. The peptide mixtures were separated using a 1-h gradient. The seven most intense precursor ions were sequentially fragmented in each scan cycle using higher-energy collisional dissociation (HCD) fragmentation. Acquired MS spectra were processed with MaxQuant software package version 1.6.7.0 with an integrated Andromeda search engine (Cox et al., 2011). Database search was performed against a target-decoy *Homo sapiens* database obtained from Uniprot, containing 96,817 protein entries and 286 commonly observed contaminants. Endoprotease trypsin was defined as a protease with a maximum of two missed cleavages. Oxidation of methionine and N-terminal acetylation were specified as variable modifications, whereas carbamidomethylation on cysteine was set as a fixed modification. The initial maximum allowed mass tolerance was set to 4.5 parts per million (ppm) for precursor ions and 20 ppm for fragment ions. The LFQ (Label-Free Quantification) algorithm was enabled, as well as matches between runs (Schwanhäusser et al., 2011) and LFQ protein intensities were used for relative protein quantification of three replicates. Gene Ontology (GO) Enrichment Analysis of biological processes and molecular function was conducted using ShinyGO v0.741 (Ge et al., 2020) (http://bioinformatics.sdstate.edu/go/) with Fisher’s exact test, false discovery rate (FDR) correction, and selecting a 0.05 *p*-value cut-off. ShinyGO was also used to generate the lollipop charts and the relationship networks between the enriched pathways.

### 2.5 Immunofluorescence

Cells were washed 3 times with phosphate-buffered saline (PBS) and fixed with 2% (w/v) formaldehyde and PBS for 15 min. The chambers were washed three times for 5 min in PBS. Subsequently, the permeabilization was done with 0.2% Triton X-100, PBS, and 1% BSA, and blocking with a mix of 3% (w/v) bovine serum albumin and PBS as a blocking agent for 30 min. This process was followed by incubation YB-1 (rabbit, 1:100, #ab76148, Abcam), vimentin (rabbit, 1:100, #D21H3, Cell Signaling), nestin (mouse, 1:50, #sc23927, Santa Cruz, Dallas, TX, United States), beta-tubulin (rabbit, 1:100, #2129 9F3, Cell Signaling, Danvers, MA, United States), TGM2 (rabbit, 1:100, #3557, Cell Signaling), Fascin-1 (mouse, 1:50, #sc46675, Santa Cruz), Zyxin (mouse, 1:100, #Z0377, Merck KGaA, Darmstadt, Germany), and Septin-9 (rabbit, 1:500, #NBP1-28711, Novus Biologicals, Centennial, CO,United States) primary antibodies as well as F-actin (1:100, #A12380 Alexa Fluor 596 phalloidin, Thermo Scientific) and G-actin staining (1:100, #D12371 DNase I Alexa Fluor 488 conjugate, Molecular Probes, Eugene, OR, United States) conjugated antibodies in 2.5% (w/v) bovine serum albumin in PBS overnight at 4 °C in a humidity chamber. Afterward, sections with primary antibodies were incubated with secondary antibodies (Alexa Fluor-594 goat anti-rabbit IgG, #a21429, Thermo Scientific; Alexa Fluor 488 goat anti-mouse IgG, #ab-150116, abcam; Alexa Fluor 594 goat anti-mouse IgG, #a-21429, Thermo Scientific; Alexa Fluor 488 goat anti-rabbit IgG, #ab150116, abcam) for 2 h with a dilution of 1:100 in 0.5% (w/v) bovine serum albumin at room temperature in the dark. The chambers were washed three times for 5 min each in PBS. Nuclear staining was performed with 1% (v/v) DAPI (Life Technologies, Darmstadt, Germany) 5 min prior to imaging. Fluorescence-stained tissue sections were visualized with a Carl Zeiss Observer Z1 fluorescence microscope (Carl Zeiss Microscopy, Jena, Germany) at a ×40 magnification. All stainings were repeated twice. For negative controls, we omitted primary antibodies.

### 2.6 Statistical analysis

**FIGURE 2:**
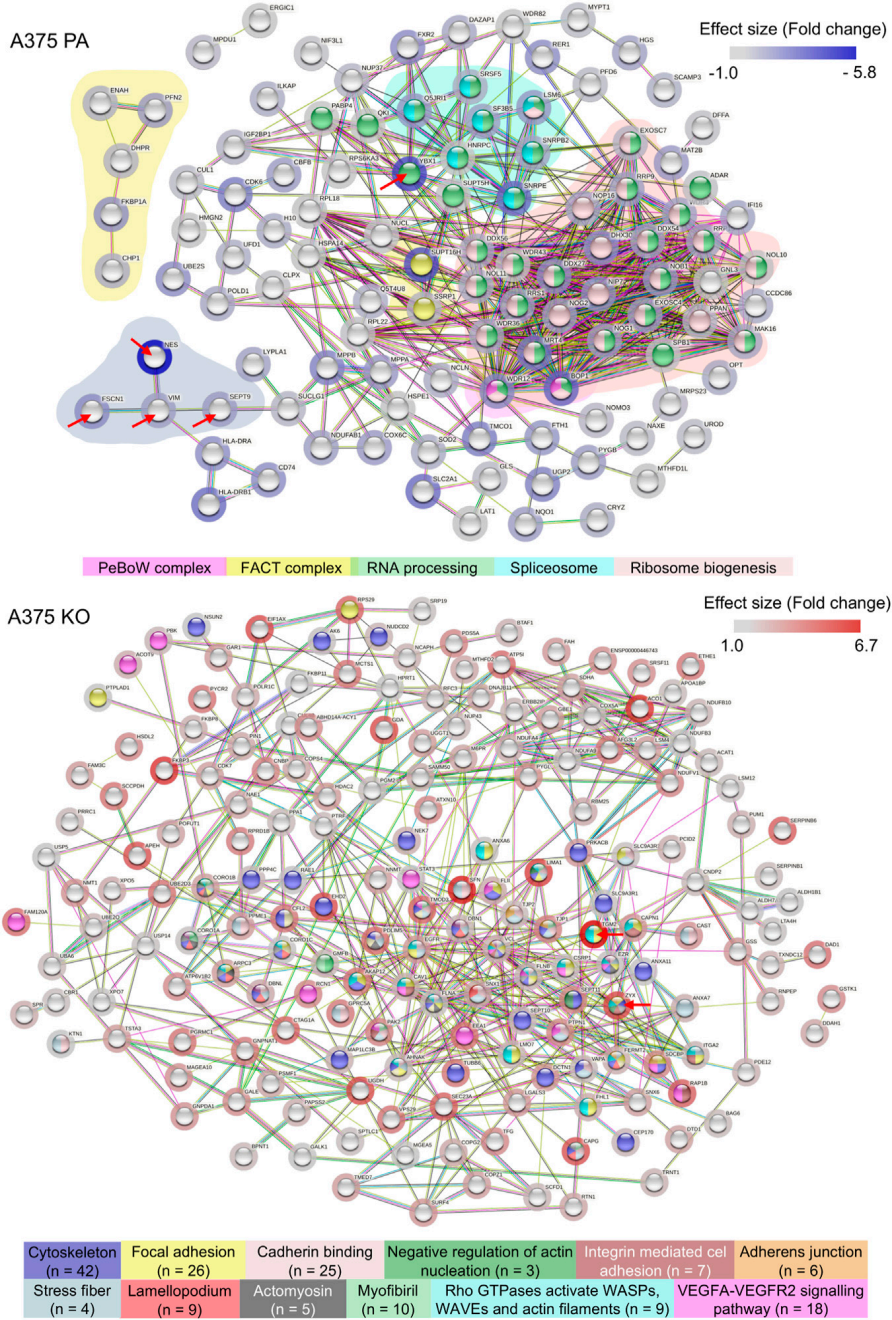
Cell stiffness as measured by Young’s modulus (kPa) is higher in YB-1 expressing parental A375 cells when compared to its YB-1 knock-out cell line. **(A)** Boxplots showing significantly higher stiffness values measured by AFM between the parental and YB-1 knock-out (*p* < 0.001), **(B)** with the descriptive statistical analysis below. *p < 0.05; Abbreviations: PA - parental; KO - knock-out; AFM - atomic force microscopy.

For further analyses, the arithmetic mean of the five AFM measurements per cell was used. Based on the non-normality of these means across the 30 measured cells per condition, AFM values are presented as median (minimum-maximum) and graphically displayed as boxplots. Descriptive statistics including standard deviation and standard error of the mean of the AFM data are additionally displayed (Figure 2B; Supplementary Figure S1). Inferential statistics were performed with the non-parametric Mann–Whitney U test to compare the differences between groups based on an alpha of 0.05. Statistical analysis was performed using the SPSS Statistics 22 (IBM Corp., Armonk, NY, United States) software.

For the proteomics data, statistical analysis was conducted using ProVision (https://provision.shinyapps.io/provision/) - an online open-source data analysis platform that allows for the analysis of MaxQuant files (Gallant et al., 2020). Briefly, reverse database hits, contaminants, and proteins identified by site modifications were removed. The file was further filtered for each protein group to contain at least two unique peptides. The assigned LFQ intensity values were subsequently log_2_ transformed to gain a normal distribution and further filtered for two values in at least one group. This resulted in a high confidence expression dataset and missing values were imputed from a truncated normal distribution of transformed LFQ intensities. Quantile plots were done within the ProVision application to check for data normality prior to statistical testing. Statistical significance was determined by an unpaired two-tailed Student’s t-test (*p* > 0.05). Multiple hypothesis testing was corrected using the Benjamini–Hochberg (FDR) set at 0.05, and a one-fold cut-off was implemented.

### 2.7 In-silico analysis

The volcano plot and clustered heatmap were generated using ProVision (https://provision.shinyapps.io/provision/). The protein-protein interaction analyses and enrichment analysis were performed using the STRING database (https://string-db.org/) by submitting the proteins with ranks (using the effect size) to normal gene set analysis and performing the Functional Enrichment Analysis with standard settings (medium confidence 0.4). The functional enrichment analysis and visualization of gene ontology (GO)3 Molecular function 2021 and GO Molecular component 2021 was performed using the Enrichr web tool (https://maayanlab.cloud/Enrichr/) (Chen et al., 2013; Kuleshov et al., 2016). Kyoto Encyclopedia of Genes and Genomes (KEGG) pathway analysis and visualization was conducted using ShinyGO v0.741 (Ge et al., 2020) (http://bioinformatics.sdstate.edu/go/) with Fisher’s exact test, false discovery rate (FDR) correction, and selecting a 0.05 *p*-value cut-off.

## 3 Results

### 3.1 YB-1 renders cells stiffer

A total of 30 cells per cell line/condition were subjected to stiffness measurements in the perinuclear region *via* AFM, and each individual cell was automatically measured five times in three independent repetitions. Thus, a total of 1350 measurements were conducted (for each cell line/condition a total of 450 measurements). The measured cellular elastic moduli are depicted in Figure 2 for the A375 cell line (YB-1 knock-out and parental). Stiffness for the A375 parental cell line with endogenous YB-1 expression was significantly higher (*p* < 0.001) than for A375 YB-1 knock-out (Figure 2). Overall, absolute values were thereby reduced by 34% with a median of 1.16 kPa for A375 parental cells to a median of 0.76 kPa for A375 knock-out cells. Interestingly, we also observed different phenotypes in A375 parental and knock-out cells (Figure 1) where parental cells were more rounded and bigger while knock-out cells were smaller and more elongated (see also Figure 1A). To account for the possible heterogeneity of mechanical properties in tumor cells, a MelJuso cell line was concomitantly subjected to AFM stiffness measurements (Supplementary Figure S1). A significant stiffness decrease was noted when comparing the two YB-1 expressing cell lines: A375 and MelJuso, with absolute values reduced by 27% (Supplementary Figure S1). This complements the view of cancer as a heterogeneous pathology, showing differences in the mechanical properties of cancerous tumors.

### 3.2 Proteomic analysis of the A375 melanoma cell line response to a YB-1 knock-out

**FIGURE 3:**
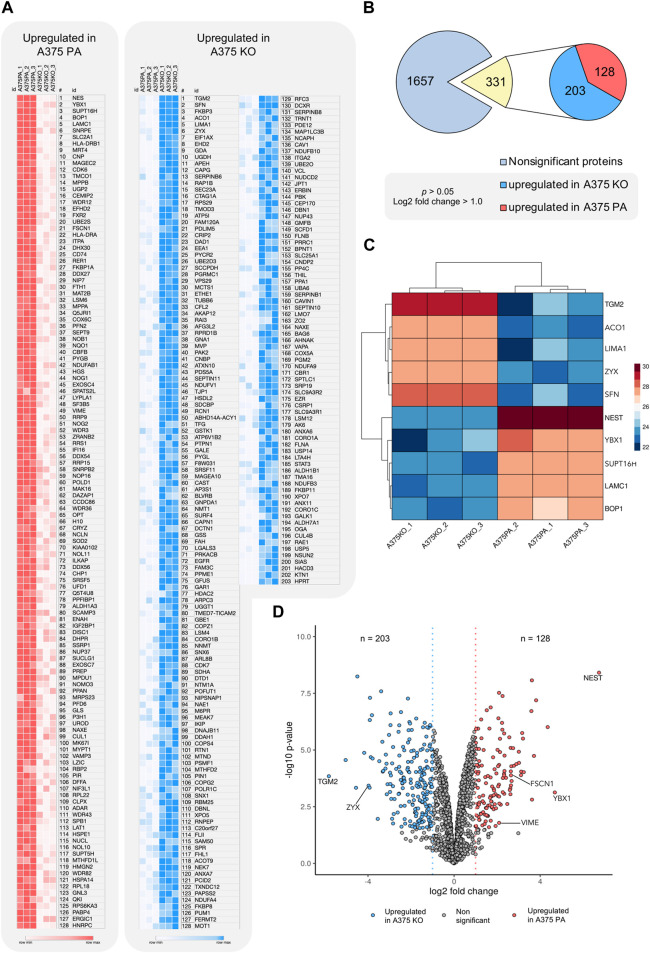
Visualization of significantly upregulated proteins in A375 parental and A375 YB-1 knock-out cell lines (*p* > 0.05, cutoff log_2_ fold change >1.0) **(A)** A heatmap of log_2_ transformed and imputed LFQ intensity values in A375 parental and A375 YB-1 knock-out (3 replicates per cell line) showing a complete list of significantly upregulated proteins in both cell lines (*p* > 0.05). Proteins are sorted according to their size effect. Generated in Morpheus Matrix Visualization (https://software.broadinstitute.org/morpheus/). **(B)** Numerical visualization of the total number of proteins identified in the proteomic analysis and the number of significantly regulated proteins. Out of 1657 proteins, statistical analysis revealed the total number of 331 significantly regulated proteins, of which 128 were significantly upregulated in A375 parental and 203 in A375 YB-1 knock-out cell line. **(C)** Clustered heatmap (complete method) of top 5 significantly upregulated proteins in both cell lines showing the proteins with the highest magnitude of expression compared to the other cell line. Columns in each cell line represent triplicates of log_2_ transformed and imputed LFQ intensity values. Rows show the expression intensity per each protein in both cell lines. Analyzed and visualized in ProVision (https://provision.shinyapps.io/provision/). **(D)** The volcano plot visualizes all proteins included in the proteomic analysis based on *p*-value and log_2_ fold change value. Each dot represents one protein. Vertical lines represent the fold change cut-off of 1.0. The proteins with the highest and lowest log_2_ fold change (*y*-axis) represent the most upregulated proteins in each cell line. Selected proteins were annotated. Analyzed and visualized in ProVision (https://provision.shinyapps.io/provision/). Abbreviations: LFQ—Label-Free Quantification; PA—parental; KO—knock-out.

The proteomic analysis aimed to further identify the main proteins and protein networks associated with the expression of YB-1 and their relation to the biomechanical and biochemical aspects of the A375 melanoma cell line with a particular focus on cytoskeleton proteins involved in EMT or ameboid migration. This analysis allowed us to reveal potential biochemical changes and signaling processes when YB-1 is present and absent. Using LFQ data, *t*-test analysis as described in section 2.6 in the MaxQuant using log 1.0-fold change cut-off revealed 331 out of 1657 significantly regulated proteins. 128 of those were upregulated in A375 parental line. 265 proteins were upregulated in A375 YB-1 knock-out cell line. Upregulation refers to the highest effect size (fold_2_ change) relative to the other cell line, which highlights the expressional changes in the presence and absence of YB-1 in a melanoma cell line. The complete list of significantly regulated proteins in parental and YB-1 knock-out cell lines sorted based on their effect size and compared to the other cell line is visualized in Figures 3A,B. They are sorted according to their effect size (fold_2_ change). Proteins with the largest difference in expression (the most upregulated in 1 cell line and absent, or nearly absent in the other cell line) are presented in the clustered heatmap in Figure 3C. Among the top 5 upregulated proteins that were also absent, or nearly absent in A375 parental cell line are (in order) nestin (NES), YB-1 (YBX1), FACT complex subunit SPT16 (SUPT16H), Ribosome biogenesis protein BOP1 (BOP1), and Laminin Subunit Gamma 1 (LAMC1) (Figure 3C). In A375 YB-1 knock-out cell line, the top 5 upregulated proteins were (in order) Protein-glutamine gamma-glutamyltransferase 2 (TGM2), 14-3-3 Protein Sigma/Stratifin (SFN), Aconitase 1 (ACO1), LIM domain and actin-binding protein 1 (LIMA1), and zyxin (ZYX) (Figure 3C). Interestingly, in YB-1 melanoma knock-out cell line, nestin was nearly absent and vimentin was expressed at significantly lower levels (Figure 3A). It has been previously shown that vimentin expression level was not altered by nestin knock-out (Yamagishi et al., 2019), which implicates YB-1 involvement in vimentin expression as a transcription factor and translational regulator. Relevant proteins related to cytoskeleton upregulated in both cell lines were marked in the volcano plot (Figure 3D). In A375 parental cell line, these are YB-1 (YBX1), vimentin (VIME), fascin-1 (FSCN1), septin-9 (SEPT9), profilin-2 (PFN2), and nestin (NES). In A375 YB-1 knock-out cell line these are transglutaminase (TGM2), Coronin 1B (Coro1B), Vinculin (VCL) and zyxin (ZYX), ezrin (EZR), and Ezrin-Radixin-Moesin Binding Phosphoprotein-50 (SLC9A3R1).

### 3.3 YB-1 knock-out induces strong upregulation of cytoskeleton proteins

**FIGURE 4:**
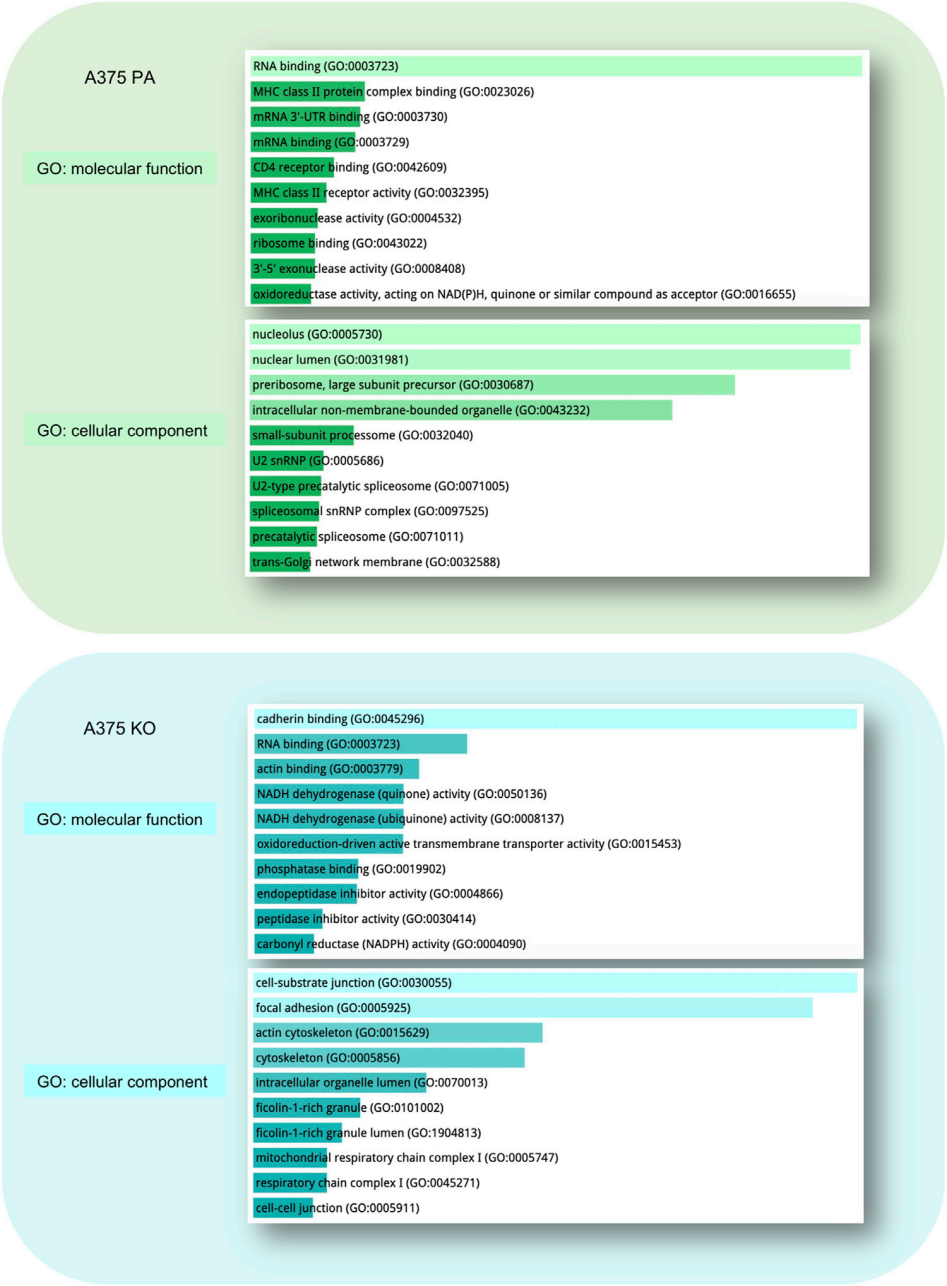
STRING analysis showing the significantly upregulated functional and physical interaction protein networks in the A375 parental and A375 YB-1 knock-out cell lines. Red arrows point to proteins that were stained with immunolabelling. The background coloring in A375 parental cell line highlights the clustering within the network. Halo color represents the effect size. The disconnected nodes were removed. Abbreviations: PA—parental; KO—knock-out.

**FIGURE 5:**
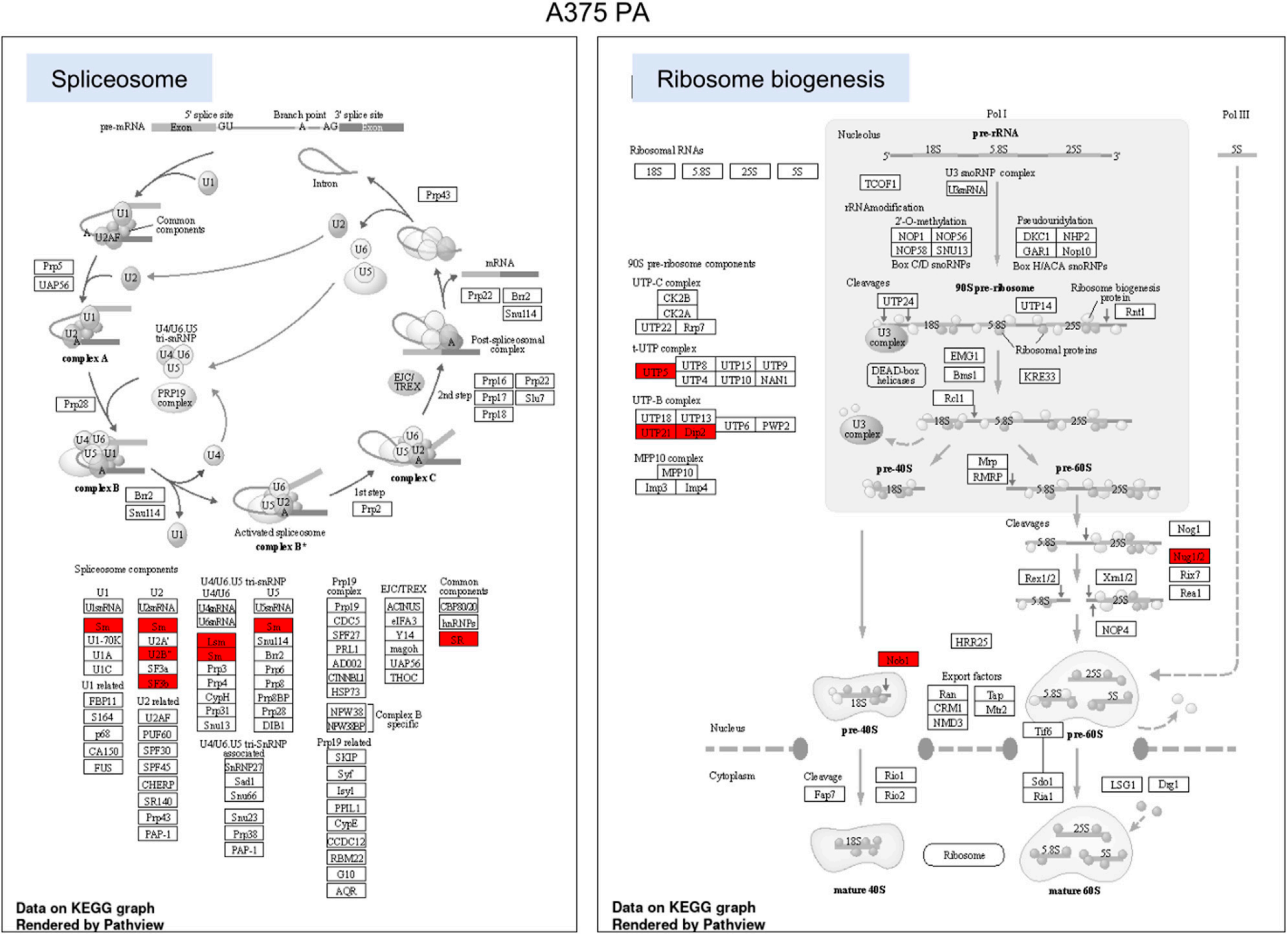
Gene Ontology (GO) Enrichment Analysis of significantly upregulated proteins in A375 parental ad A375 YB-1 knock-out cell lines. The analysis and visualization were performed in Enrichr web (https://maayanlab.cloud/Enrichr/) (Chen et al., 2013; Kuleshov et al., 2016). The list of upregulated proteins for each cell line was used to generate the results. The top enriched molecular function and cellular components are visualized for each cell line. All results are sorted by *p*-value ranking. Abbreviations: GO—Gene Ontology, PA—parental; KO—knock-out.

**FIGURE 6:**
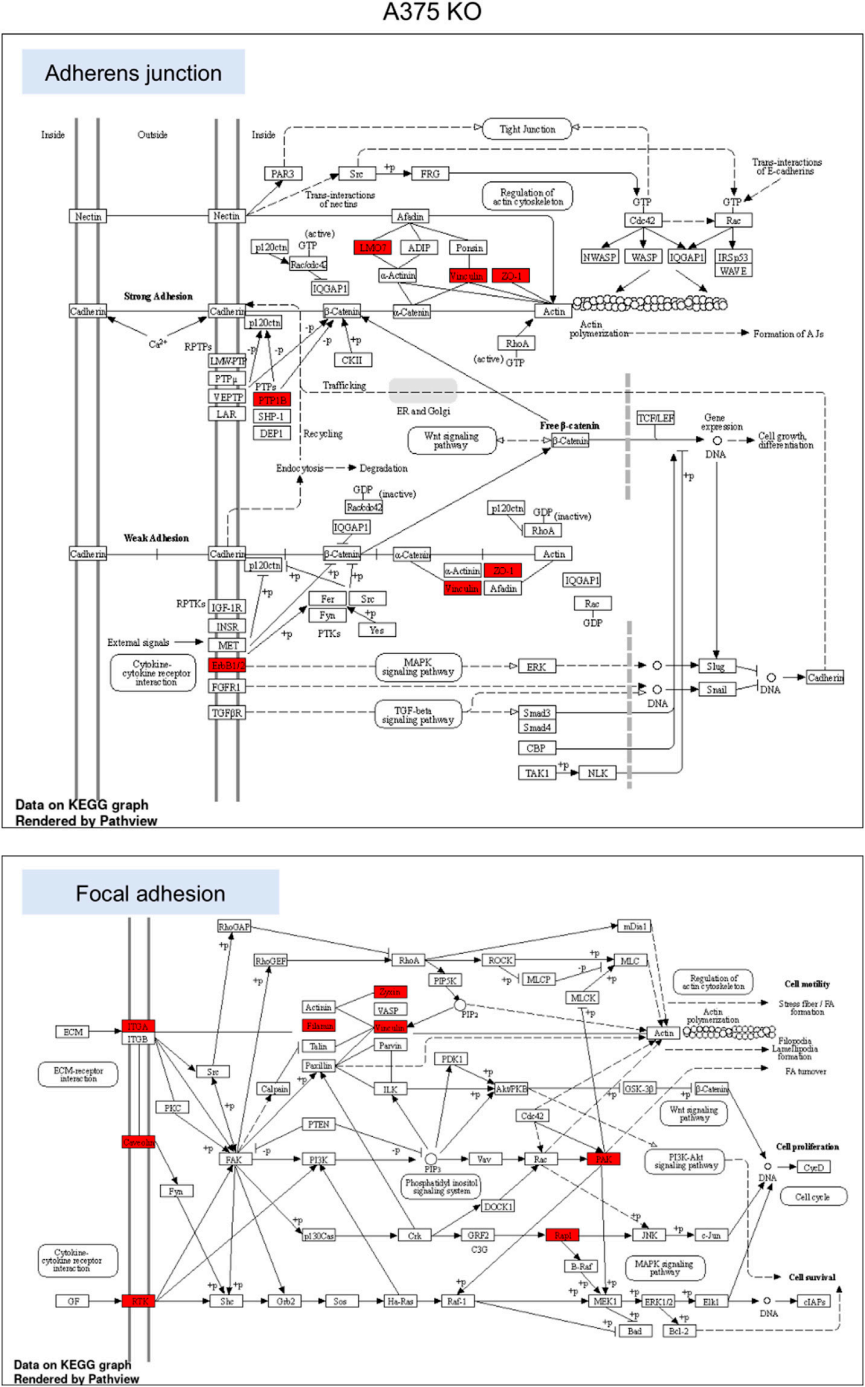
KEGG pathway enrichment analysis in A375 parental cell line. Red represented high expression. Abbreviations: KEGG–Kyoto Encyclopedia of Genes and Genomes, PA—parental; KO—knock-out. Figure 6. KEGG pathway enrichment analysis in A375 YB-1 knock-out cell line. Red represented high expression. Abbreviations: KEGG–Kyoto Encyclopedia of Genes and Genomes, PA—parental; KO—knock-out.

**FIGURE 7:**
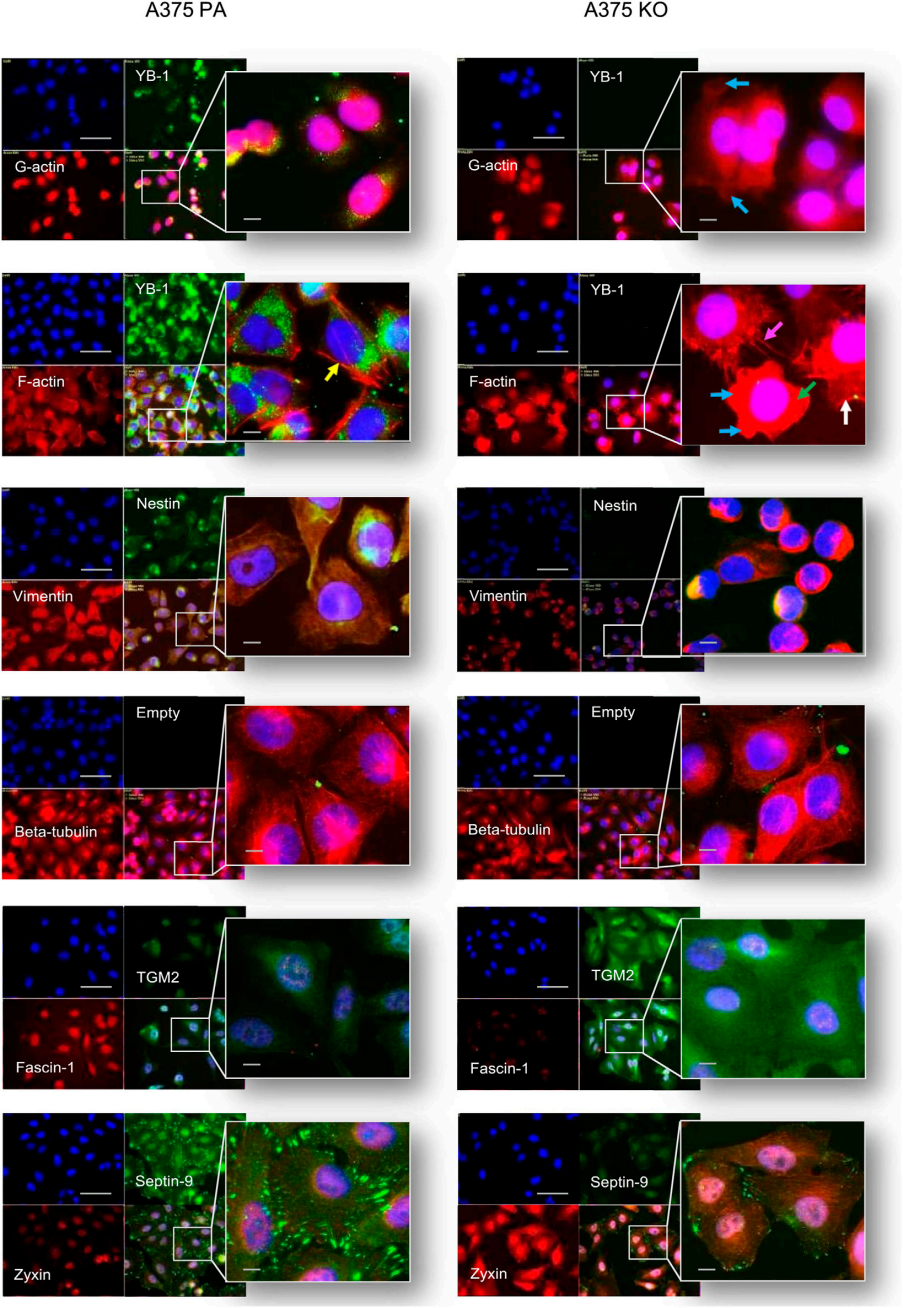
Immunolabelling of cytoskeleton protein in A375 parental and A375 YB-1 knock-out cell lines. From top to bottom, co-labeling of: YB-1 - green, F-actin - red, nuclei - blue; vimentin - red, nestin - green, nuclei—blue; beta-tubulin- red, nuclei-blue; fascin-1 - red, TGM2 - green, nuclei-blue; zyxin—red, septin-9 - green, nuclei-blue, were conducted for these 2 cell lines A375 parental (left side) and A375 knock-out (right side). Arrows denote morphological differences: loss of F-actin (marked by green arrows), blebbing (marked by the blue arrow), filopodia formation (marked by magenta arrow)., lamellipodia (marked by white arrows), stress (marked by yellow arrows). Alexa 488 - green labeling, Alexa 594 - red labeling, DAPI - blue (nucleus staining). The scale bar (white) represents 20 µm. Abbreviations: PA - parental; KO - knock-out.

To understand global biochemical and cell signaling changes occurring in the cell when YB-1 is present and absent, we performed the functional and enrichment analysis at three levels: we first examined the functional and physical protein interactions using STRING (Figure 4). Secondly, we used gene ontology (GO) classification of molecular functions and cellular components to analyze the properties of genes and gene products from different perspectives (Figure 5). Thirdly, we explored KEGG pathways to examine the key upregulated signaling pathways in both cell lines (Figure 6 and Figure 7). As the input for STRING analysis, we used a ranked list (based on calculated effect size from the *t*-test) of significantly regulated proteins in parental and YB-1 knock-out cell lines. In A375 parental cell line, STRING analysis showed protein-protein interaction (PPI) enrichment *p*-value <1.0e-16, meaning that means that proteins have more interactions among themselves than what would be expected for a random set of proteins. The protein network revealed distinct clusters, highly interconnected. Functional analysis in STRING demonstrated strong upregulation of whole clusters of proteins involved in ribosome biogenesis, RNA processing, RNA maturation, RNA binding, protein folding, ribosome biogenesis, ubiquitination, and spliceosome. Most proteins co-shared functions, therefore for the purpose of the analysis, we only marked the most functionally distinct, functionally enriched clusters. Although it was not functionally enriched, the interactions revealed the physical and direct network of nestin, fascin-1, vimentin, and septin-9, all strongly upregulated in A375 parental cell line. Out of 128 proteins, only 7 could be linked to the cytoskeleton, profilin-2 (PFN2), CD63, Protein enabled homolog (ENAH), vimentin (VIM), septin-9 (SEPT9), and fascin-1 (FSC1). In contrast, the number of proteins involved in RNA maturation, binding, and biogenesis was 62. In addition, the enrichment analysis revealed strong expression of molecular components present in FACT and PeBoW complexes. The FACT complex plays a critical role in transcription initiation, transcription elongation (Grasser, 2020), DNA replication, and DNA repair while PeBoW complex is essential for ribosome biogenesis and cell cycle progression. The control of ribosome biogenesis is a critical cellular nodal point and the deregulation of ribosomes may cause carcinogenesis (Finkbeiner et al., 2011). In A375 YB-1 knock-out cell line the PPI enrichment *p*-value was also less than 1.0e-16. In YB-1 knock-out, surprisingly, the most upregulated proteins were related to actin filament, stress fibers, cytoskeleton, focal adhesion, actomyosin, cadherin binding, integrin-mediated cell adhesion, focal adhesion., VEGF-VEGFR2 signaling pathway, or lamellipodium formation. Out of 203 proteins, 42 were involved in cytoskeleton organization, which contrasted with A375 parental line where only 7 proteins could be linked to the cytoskeleton. Those proteins were well interconnected within the network, without any distinct and functionally separated clusters suggesting global and organized changes within the cell focused on the cytoskeleton. Significantly upregulated proteins that were also functionally enriched are described in Supplementary Table S1 (A375 parental cell line) and Supplementary Table S2 (A375 YB-1 knock-out).

Gene ontology (GO) of the top 10 most enriched molecular functions and cellular components in each cell line is visualized in Figure 5. In parental cell line, the key molecular functions were related to RNA binding and the top cellular components included nucleolus, nuclear lumen, preribosomes, and intracellular non-membrane-bounded organelle. In A375 YB-1 knock-out, the key molecular functions were related to the cell-substrate junction, focal adhesion, and cytoskeleton while the most highly enriched cellular component was cadherin binding, RNA binding, and actin binding.

To highlight the upregulated signaling pathways induced by the YB-1 we used the two top enriched KEGG pathways and visualized (in red) the upregulated components within the pathway. In YB-1 expressing, A375 parental cell line, the two key pathways were related to spliceosome and ribosome biogenesis (Figure 6). In A375 YB-1 knock-out cell line the two key pathways were adherens junction and focal adhesion (Figure 7).

### 3.4 Qualitative assessment and localization of cytoskeleton protein expression

To investigate the relationship between the biochemical and biomechanical changes as well as the cellular localization of selected proteins in YB-1 expressing and non-YB-1 expressing cell lines, we used immunofluorescence (IF). Our selected targets corresponded to the cytoskeleton proteins of interest, based on both the current literature and the results of the proteome analysis. Our immunofluorescence analysis confirmed the presence and stability of the knock-out, whereby YB-1 is strongly expressed in A375 parental cell line and completely absent in A375 knock-out. These results are in line with those of Kosnopfel et al., confirming that the CRISPR-based knock-out technique of YB-1 in A375 melanoma cells renders a stable loss of protein expression following genetic changes (Kosnopfel et al., 2018). With respect to immunolabelling of cytoskeleton proteins, G-actin (globular) and F-actin (filamentous) were co-stained with YB-1. In YB-1 expressing cell line, G-actin was strongly and preferentially expressed in the nuclear region. In contrast, G-actin was widely expressed in both the nucleus and the cytoplasm of YB-1 knock-out. F-actin is the main driver of cell motility during mesenchymal and ameboid migration (Izdebska et al., 2020). Arrows highlight striking differences in F-actin organization in A375 parental and YB-1 knock-out cell lines. In A375 parental cell line, F-actin filaments are thin and elongated. In A375 YB-1 knock-out cell line the difference between G-actin and F-actin is less evident. In some cells, filamentous actin stress fibers are completely lost (marked by green arrows, Figure 6) which happens during mesenchymal to ameboid transition. Some cells present a “blebbing” phenotype which is also a hallmark of ameboid migration (Holle et al., 2019) (marked by the blue arrow, Figure 6). Notably, not all cells in A375 YB-1 knock-out present the same phenotype. In some there is visible filopodia formation (marked by the magenta arrow, Figure 6)., in others, lamellipodia are more pronounced (marked by white arrows, Figure 6). In contrast, in A375 parental cell line, cells are relatively consistent in their F-actin organization. In YB-expressing cell line, stress fibers are clearly visible in all cells (marked by yellow arrows, Figure 6). Both vimentin and nestin were abundant in the cytoplasm and nucleus of the parental cell line. Vimentin expression is associated with more mesenchymal cell shape and increased focal adhesion formation which is structurally connected to stress fibers (Ostrowska-Podhorodecka et al., 2022). In A375 parental cell line, we observed a very strong expression of vimentin, which correlates with the presence of F-actin stress fibers. In A375 YB-1 knock-out cell line, vimentin was expressed solely perinuclearly, whereas nestin appeared to be completely absent. This observation was also consistent with the proteomics results where LFQ data showed that nestin was nearly absent in the knock-out cell line. The labeling of another important cytoskeleton component - beta-tubulin showed that the tubulin network is strongly expressed in both cell lines, however, in parental cell line, there were notably richer perinuclear networks than the knock-out counterparts. TGM2 had a weak signal in the parental cell line but was strongly and ubiquitously expressed in the YB-1 knock-out. TGM2 signal was the strongest perinuclearly. In the case of fascin-1, it was strongly expressed in the nuclear area of the A375 parental cell line, but it showed a weak signal in the YB-1 knock-out cells. The latter was also consistent with the quantitative proteomics analysis results. Septin-9 was strongly expressed in parental cell lines, revealing a well-interconnected network of filaments concentrated at the cell boundaries. In contrast, septin-9 expression in YB-1 knock-out was very weak with notably less prominent filaments. Zyxin immunolabelling exhibited a notably weaker and less prevalent signal in the parental cell. Contrastingly, in YB-1 knock-out cells, zyxin was strongly expressed in the nucleus as well as in the cytoplasm with visible string networks observed.

## 4 Discussion

Malignant tumors have a high capacity to adapt to the selective pressures they encounter at every stage of tumor development, from early stages of tumor initiation, through disease progression, as well as in response to therapy. Signaling plasticity is utilized for a variety of cancer mechanisms such as invasiveness and cell migration, including aspects of mesenchymal and amoeboid migration. Melanomas are highly metastatic skin tumors that use both modes of migration. Y-box binding protein 1 (YB-1) is an important oncogenic protein, overexpressed in malignant melanoma with increasing protein levels during tumor progression (Sinnberg et al., 2012; Kosnopfel et al., 2018). Previous consistently showed that in malignant cancers extracellular YB-1 promotes the translation of mRNAs encoding proteins involved in epithelial-to-mesenchymal transition (EMT) melanoma migration, invasion, and *in vitro* tumorigenicity (Evdokimova et al., 2009; El-Naggar et al., 2015). YB-1 secretion is associated with poor prognosis highlighting an important role of YB-1 in melanoma development and progression (Evdokimova et al., 2009; Khan et al., 2014; Kosnopfel et al., 2018). YB-1 impact on migration signaling pathways in the context of melanoma metastases and how these signaling pathways become hyperactivated in invasive phenotypes are not fully understood (de Winde et al., 2021). While EMT is a well-described process, ameboid migration is not yet fully understood. Using the knock-out model, we aimed to elucidate biochemical signaling as well as biomechanical properties of the melanoma cells in relation to the biochemical composition of the cytoskeleton. According to our proteomic results, in YB-1 expressing melanoma cell line, the highly upregulated proteins were strongly related to ribosomes, mRNA, and spliceosome, which is in agreement with previous studies elucidating the role of cytoplasmic YB-1 as is a part of messenger ribonucleoprotein particles (mRNPs) and a controller of translation and stability of mRNA (Evdokimova et al., 2001; Mordovkina et al., 2020). What was interesting is that YB-1 expression induced a very strong expression of intermediate filaments (IF) - nestin (6-fold change) and vimentin (2-fold change) while YB-1 knock-out induced complete depletion of nestin and significantly lower expression of vimentin. Vimentin is a canonical marker of EMT (Satelli and Li, 2011) and its high expression, similarly to YB-1, has been shown to induce a highly motile, invasive phenotype in melanoma. Previous studies demonstrated that nestin knock-out does not affect vimentin expression (Yamagishi et al., 2019), which suggests that YB-1 plays some role in the upregulation of both nestin and vimentin, which are both highly relevant prognostic markers of malignancy in melanoma. Interestingly, previous research showed that while nestin is strongly upregulated in invasive melanoma, nestin depletion also enhances melanoma invasion *in vitro* (Lee et al., 2014). Vimentin has been previously implicated in playing a crucial role in maintaining cytoskeleton architecture and cellular mechanical strength. Vimentin intermediate filaments (IF) exhibit distinct viscoelastic properties that are not exhibited by other filamentous biopolymers such as actin filaments (Shah et al., 1998). Vimentin deficient fibroblasts are more compliant and less motile (Wang and Stamenović, 2000), which could explain lower cell stiffness in YB-1 knock-out cell line where vimentin expression was significantly lower. Downregulation of both vimentin and nestin has been previously linked to drug resistance in melanoma, as well as upregulation of processes such as PI3K/AKT signaling, remodeling of focal adhesions, actin cytoskeleton, and integrin signaling (Schmitt et al., 2019).

**FIGURE 8:**
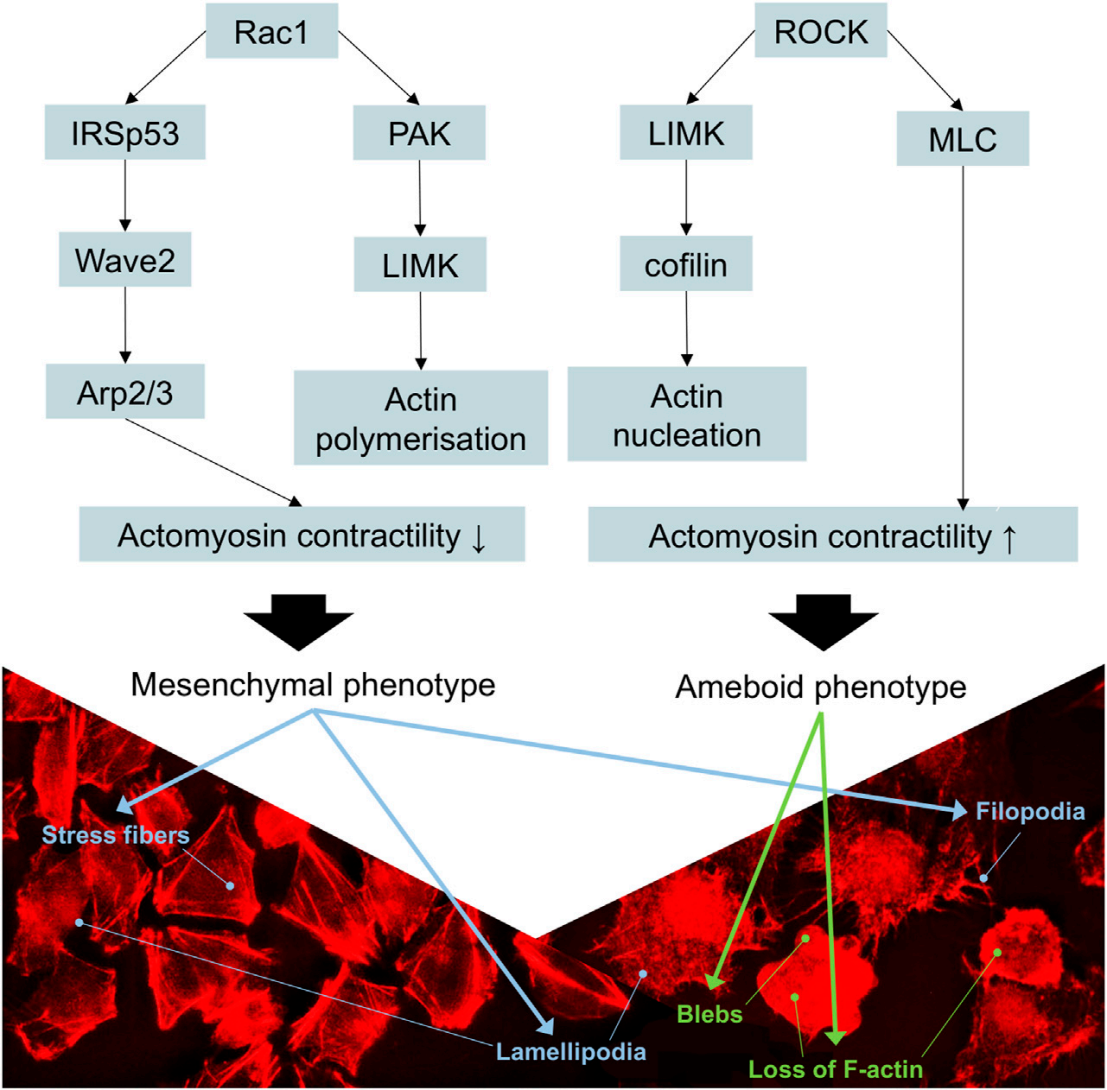
Schematic representation of the signaling cascades involved in the movement of melanoma cells. Rac1 activation decreases the contractility of actomyosin, promoting mesenchymal movement, whereas the activation of RhoA/ROCK cascade increases actomyosin contractility, resulting in amoeboid movement. p21-activated kinase (PAK) binds to the GTP-bound form of Rac1, activating the LIM-motif-containing protein kinase (LIMK) to drive cofilin-mediated actin turnover. IRSp53 (insulin receptor tyrosine kinase substrate p53) is an adaptor protein that binds to Rac1 and Wave 2, thus contributing to the formation of lamellipodia. Stress fibers, lamellipodia, and filopodia are characteristic of mesenchymal phenotype. Rounded morphology, loss of actin filaments, and formation of blebs are characteristics of ameboid phenotype. Adapted from (Bonaventure et al., 2013).

Overall, the landscape of upregulated proteins in parental melanoma cell lines seemed to relate to protein turnover, ubiquitination, cell survival, proliferation, and anti-apoptotic features (which are beyond the scope of this study), as well as known markers of mesenchymal phenotype. The only cytoskeleton proteins not mentioned before that were upregulated relative to YB-1 knock-out were profilin-2 (PFN2), septin-9 (SEPT9), and fascin-1 (FSC1). Profilin-2, as a downstream regulator of TGF-β/Smad signaling, plays a pro-tumoral role linked to EMT processes in colorectal cancer (Kim et al., 2015). Septins are commonly required for mesenchymal migration, while fascin-1 (Liu et al., 2021) overexpression is involved in EMT. In previous studies, YB-1 downregulation resulted not only in a reduced proliferation rate of melanoma cells but also in a higher number of apoptotic cells and increased sensitivity to chemotherapy (Schittek et al., 2007). In our study, complete YB-1 knockout induced upregulation of a significant amount of proteins involved in pathways leading to increased invasiveness and motility. The network of significantly upregulated proteins was very interconnected, without distinct, disconnected clusters as in YB-1 expressing melanoma cell line, suggesting organized overexpression of proteins involved in the cytoskeleton, focal adhesion, and adherens junction (Figure 4). Immunolabelling of F-actin in YB-1 knock-out cell line demonstrated a spectrum of mesenchymal phenotypes, including lamellipodia, or filopodia. We found strong upregulation of coronins, including Coronin 1B (Coro1B) which is actin binding protein that controls actin networks at classical lamellipodia (Werner et al., 2020). Vinculin and zyxin are adaptor proteins involved in forming more stable focal adhesions (Panková et al., 2010), while tissue transglutaminase (TGM2) promotes adhesion, spreading of cells, and enhances focal adhesions which are crucial to mesenchymal migrating cells (Fok et al., 2006). With immunolabelling, we also demonstrated that YB-1 knock-out melanoma cell line showed important hallmarks of ameboid migration such as blebbing and loss of actin filaments. Such phenotype would be consistent with the upregulation of important proteins involved in blebbing such as ezrin (EZR) and Ezrin-Radixin-Moesin Binding Phosphoprotein-50 (SLC9A3R1). Ameboid migration is attributed to the RhoA-ROCK signaling axis (Figure 8) which increases actomyosin contractility to generate membrane blebs, reinforced by JAK- STAT3 signaling. STAT3 has been previously associated with a round morphology and amoeboid-type movement. In our study, it was also found to be significantly upregulated in YB-1 knock-out melanoma cell line. This strengthens our hypothesis that global proteome changes in YB-1 knock-out cell population appear to show signs of both mesenchymal and ameboid migration, as the transition is likely to be a continuous gradient, not a binary switch as shown in previous research. Previous studies have shown that melanoma cells adopt features of both mesenchymal and amoeboid migration (Gabbireddy et al., 2021), and our study confirms this observation.

Ameboid phenotype is softer and more deformable (Panková et al., 2010) which together with the depletion of IF such as nestin and vimentin could explain why YB-1 knockout melanoma cell line was more elastic (less stiff) compared to parental, YB-1 expressing cell line. In this study, we have demonstrated that melanoma is a very plastic and adaptable tumor entity, able to simultaneously express features of mesenchymal and ameboid phenotype and can easily compensate and switch alternative signaling pathways to restore invasive properties (Kashani and Packirisamy, 2020). Stiffness has been long hypothesized to be a potential biomarker of metastatic potential and several *in vitro* studies reported that higher cell stiffness may indicate lower metastatic potential (Watanabe et al., 2012), however, our study warrants caution when using stiffness as an indicator of metastatic potential. Phenotype seems to be more important in melanoma rather than stiffness alone, as it is related to the expression of multitude of proteins in response to the environment, therefore, our study confirms that it is unlikely that stiffness alone will be a reliable indicator of metastatic potential. Melanoma cancer cells are able to fluctuate within the spectrum of migratory modes which results in stiffness alteration. In addition, those changes can occur within hours depending on the environmental cues. Our study demonstrated that constitutive expression of YB-1 in melanoma seems to be tightly related to both nestin and vimentin expression which results in a stiffer phenotype, as well as a wide array of proteins involved in RNA, ribosomes, and spliceosome in A375 melanoma cell line. We found, that YB-1 knock-out resulted in nestin depletion and significantly lower vimentin expression. Moreover, YB-1 knock-out induced global cellular changes focused on cytoskeleton and migration, which could be an attempt to compensate for the lack of YB-1 resulting in a strong overrepresentation of pathways signaling for mesenchymal/ameboid migration.

To date, this is the first study to specifically investigate the role of YB-1, a known EMT inducer and biomarker of metastatic potential in melanoma cells. Our study explores the protein landscape and signaling changes involved in the switch from ameboid to mesenchymal phenotype which may contribute to the identification of potential biomarkers, allowing to predict amoeboid invasion and metastatic progression. Our proteomic study demonstrates that signaling pathways involved in ameboid to mesenchymal phenotype are very plastic and adaptable and gives insight into the implication of YB-1 expression on potential migratory phenotypes and signaling pathways. As YB-1 expression predicts drug resistance and patient outcome in numerous tumors, our findings in YB-1 knock-out model are of relevance and warrant further studies.

## 5 Conclusion

Expression of YB-1 regulates complex and intrinsic networks and cellular pathways which overall render cells stiffer in A375 melanoma cell line. YB-1 knock-out induces global cellular upregulation of proteins related to cytoskeleton, including features of both mesenchymal and ameboid migration. Melanoma is a highly plastic, adaptable, and aggressive tumor entity, able to exhibit characteristics of different migratory modes.

## 6 Study limitations

While our study provides an intriguing new perspective on the plasticity of melanoma and the cellular pathways which render cells stiffer, the methods used imply arduous work. As a result, only three independent experiments for each cell line were eventually carried out. This should be considered when interpreting statistical results, but the measured tendency should not be affected. Furthermore, cellular heterogeneity in tumor cells is a well-established phenomenon (Huxley, 1958; Alizadeh et al., 2015) that is thought to be an important cause of drug resistance that impedes treatment outcome Although our study only investigated one melanoma cell line, which may not be representative of the entire neoplastic cell population, our findings are highly consistent with previous studies that looked at and found a high biochemical and biomechanical plasticity of melanoma (Weder et al., 2014; Schmitt et al., 2019; Avagliano et al., 2020). Finally, despite the fact that CRISPR/Cas9 technology has become the most studied gene editing technology in recent years due to its simple design, it still faces numerous challenges, such as the occurrence of off-targeting modifications, which cannot always be predicted in advance.

## Data Availability

Existing datasets are available in a publicly accessible repository: Publicly available datasets were analyzed in this study. This data can be found here: https://www.ebi.ac.uk/biostudies/studies/S-BSST1067.

## References

[B1] Alizadeh A. A. Aranda V. Bardelli A. Blanpain C. Bock C. Borowski C. (2015). Toward understanding and exploiting tumor heterogeneity. Nat. Med. 21 (8), 846–853. 10.1038/nm.3915 26248267 PMC4785013

[B2] Avagliano A. Fiume G. Pelagalli A. Sanità G. Ruocco M. R. Montagnani S. (2020). Metabolic plasticity of melanoma cells and their crosstalk with tumor microenvironment. Front. Oncol. 10, 722. 10.3389/fonc.2020.00722 32528879 PMC7256186

[B3] Basaki Y. Taguchi K. Izumi H. Murakami Y. Kubo T. Hosoi F. (2010). Y-box binding protein-1 (YB-1) promotes cell cycle progression through CDC6-dependent pathway in human cancer cells. Eur. J. cancer 46 (5), 954–965. 10.1016/j.ejca.2009.12.024 20079629

[B4] Bonaventure J. Domingues M. J. Larue L. (2013). Cellular and molecular mechanisms controlling the migration of melanocytes and melanoma cells. Pigment Cell and Melanoma Res. 26 (3), 316–325. 10.1111/pcmr.12080 23433358

[B5] Borchert N. Dieterich C. Krug K. Schütz W. Jung S. Nordheim A. (2010). Proteogenomics of Pristionchus pacificus reveals distinct proteome structure of nematode models. Genome Res. 20 (6), 837–846. 10.1101/gr.103119.109 20237107 PMC2877580

[B6] Chan E. Saito A. Honda T. Di Guglielmo G. M. (2014). The acetylenic tricyclic bis(cyano enone), TBE-31 inhibits non-small cell lung cancer cell migration through direct binding with actin. Cancer Prev. Res. 7 (7), 727–737. 10.1158/1940-6207.CAPR-13-0403 24806663

[B7] Charras G. Paluch E. (2008). Blebs lead the way: How to migrate without lamellipodia. Nat. Rev. Mol. Cell Biol. 9 (9), 730–736. 10.1038/nrm2453 18628785

[B8] Chen E. Y. Tan C. M. Kou Y. Duan Q. Wang Z. Meirelles G. V. (2013). Enrichr: Interactive and collaborative HTML5 gene list enrichment analysis tool. BMC Bioinforma. 14 (1), 128. 10.1186/1471-2105-14-128 PMC3637064 23586463

[B9] Cox J. Neuhauser N. Michalski A. Scheltema R. A. Olsen J. V. Mann M. (2011). Andromeda: A peptide search engine integrated into the MaxQuant environment. J. proteome Res. 10 (4), 1794–1805. 10.1021/pr101065j 21254760

[B10] de Winde C. M. George S. L. Crosas-Molist E. Hari-Gupta Y. Arp A. B. Benjamin A. C. (2021). Podoplanin drives dedifferentiation and amoeboid invasion of melanoma. iScience 24 (9), 102976. 10.1016/j.isci.2021.102976 34485858 PMC8405990

[B11] Eitaki M. Yamamori T. Meike S. Yasui H. Inanami O. (2012). Vincristine enhances amoeboid-like motility via GEF-H1/RhoA/ROCK/Myosin light chain signaling in MKN45 cells. BMC Cancer 12, 469. 10.1186/1471-2407-12-469 23057787 PMC3522013

[B12] El-Naggar A. M. Veinotte C. J. Cheng H. Grunewald T. G. Negri G. L. Somasekharan S. P. (2015). Translational activation of HIF1α by YB-1 promotes sarcoma metastasis. Cancer Cell 27 (5), 682–697. 10.1016/j.ccell.2015.04.003 25965573

[B13] Evdokimova V. Ruzanov P. Imataka H. Raught B. Svitkin Y. Ovchinnikov L. P. (2001). The major mRNA-associated protein YB-1 is a potent 5' cap-dependent mRNA stabilizer. Embo J. 20 (19), 5491–5502. 10.1093/emboj/20.19.5491 11574481 PMC125650

[B14] Evdokimova V. Tognon C. Ng T. Ruzanov P. Melnyk N. Fink D. (2009). Translational activation of snail1 and other developmentally regulated transcription factors by YB-1 promotes an epithelial-mesenchymal transition. Cancer Cell 15 (5), 402–415. 10.1016/j.ccr.2009.03.017 19411069

[B15] Ferreira A. R. Bettencourt M. Alho I. Costa A. L. Sousa A. R. Mansinho A. (2017). Serum YB-1 (Y-box binding protein 1) as a biomarker of bone disease progression in patients with breast cancer and bone metastases. J. Bone Oncol. 6, 16–21. 10.1016/j.jbo.2017.01.002 28194325 PMC5294742

[B16] Finkbeiner E. Haindl M. Raman N. Muller S. (2011). SUMO routes ribosome maturation. Nucleus 2 (6), 527–532. 10.4161/nucl.2.6.17604 22064470

[B17] Fok J. Y. Ekmekcioglu S. Mehta K. (2006). Implications of tissue transglutaminase expression in malignant melanoma. Mol. Cancer Ther. 5 (6), 1493–1503. 10.1158/1535-7163.MCT-06-0083 16818508

[B18] Gabbireddy S. R. Vosatka K. W. Chung A. J. Logue J. S. (2021). Melanoma cells adopt features of both mesenchymal and amoeboid migration within confining channels. Sci. Rep. 11 (1), 17804. 10.1038/s41598-021-97348-7 34493759 PMC8423822

[B19] Gallant J. L. Heunis T. Sampson S. L. Bitter W. (2020). ProVision: A web-based platform for rapid analysis of proteomics data processed by MaxQuant. Bioinformatics 36 (19), 4965–4967. 10.1093/bioinformatics/btaa620 32638008 PMC7723325

[B20] Ge S. X. Jung D. Yao R. (2020). ShinyGO: A graphical gene-set enrichment tool for animals and plants. Bioinformatics 36 (8), 2628–2629. 10.1093/bioinformatics/btz931 31882993 PMC7178415

[B21] Grasser K. D. (2020). The FACT histone chaperone: Tuning gene transcription in the chromatin context to modulate plant growth and development. Front. Plant Sci. 11, 85. 10.3389/fpls.2020.00085 32140163 PMC7042381

[B22] Habibi G. Leung S. Law J. H. Gelmon K. Masoudi H. Turbin D. (2008). Redefining prognostic factors for breast cancer: YB-1 is a stronger predictor of relapse and disease-specific survival than estrogen receptor or HER-2 across all tumor subtypes. Breast cancer Res. BCR 10 (5), R86. 10.1186/bcr2156 18925950 PMC2614522

[B23] Hecht I. Bar-El Y. Balmer F. Natan S. Tsarfaty I. Schweitzer F. (2015). Tumor invasion optimization by mesenchymal-amoeboid heterogeneity. Sci. Rep. 5 (1), 10622. 10.1038/srep10622 26013062 PMC4650638

[B24] Holle A. W. Govindan Kutty Devi N. Clar K. Fan A. Saif T. Kemkemer R. (2019). Cancer cells invade confined microchannels via a self-directed mesenchymal-to- amoeboid transition. Nano Lett. 19 (4), 2280–2290. 10.1021/acs.nanolett.8b04720 30775927 PMC6463244

[B25] Huxley J. (1958). Biological aspects of cancer. New York: Harcourt, Brace.

[B26] Izdebska M. Zielińska W. Hałas-Wiśniewska M. Grzanka A. (2020). Involvement of actin and actin-binding proteins in carcinogenesis. Cells 9 (10), 2245. 10.3390/cells9102245 33036298 PMC7600575

[B27] Kashani A. S. Packirisamy M. (2020). Cancer cells optimize elasticity for efficient migration. R. Soc. Open Sci. 7 (10), 200747. 10.1098/rsos.200747 33204453 PMC7657900

[B28] Khan M. I. Adhami V. M. Lall R. K. Sechi M. Joshi D. C. Haidar O. M. (2014). YB-1 expression promotes epithelial-to-mesenchymal transition in prostate cancer that is inhibited by a small molecule fisetin. Oncotarget 5 (9), 2462–2474. 10.18632/oncotarget.1790 24770864 PMC4058019

[B29] Kim M.-J. Lee Y.-S. Han G.-Y. Lee H.-N. Ahn C. Kim C.-W. (2015). Profilin 2 promotes migration, invasion, and stemness of HT29 human colorectal cancer stem cells. Biosci. Biotechnol. Biochem. 79 (9), 1438–1446. 10.1080/09168451.2015.1043118 25964982

[B30] Kliza K. Taumer C. Pinzuti I. Franz-Wachtel M. Kunzelmann S. Stieglitz B. (2017). Internally tagged ubiquitin: A tool to identify linear polyubiquitin-modified proteins by mass spectrometry. Nat. methods 14 (5), 504–512. 10.1038/nmeth.4228 28319114

[B31] Kosnopfel C. Sinnberg T. Sauer B. Busch C. Niessner H. Schmitt A. (2018). YB-1 expression and phosphorylation regulate tumorigenicity and invasiveness in melanoma by influencing EMT. Mol. Cancer Res. 16 (7), 1149–1160. 10.1158/1541-7786.MCR-17-0528 29743296

[B32] Kosnopfel C. Sinnberg T. Sauer B. Niessner H. Muenchow A. Fehrenbacher B. (2020). Tumour progression stage-dependent secretion of YB-1 stimulates melanoma cell migration and invasion. Cancers (Basel) 12 (8), 2328. 10.3390/cancers12082328 32824741 PMC7464723

[B33] Kosnopfel C. Sinnberg T. Sauer B. Niessner H. Schmitt A. Makino E. (2017). Human melanoma cells resistant to MAPK inhibitors can be effectively targeted by inhibition of the p90 ribosomal S6 kinase. Oncotarget 8 (22), 35761–35775. 10.18632/oncotarget.16204 28415756 PMC5482615

[B34] Kosnopfel C. Sinnberg T. Schittek B. (2014). Y-box binding protein 1--a prognostic marker and target in tumour therapy. Eur. J. Cell Biol. 93 (1-2), 61–70. 10.1016/j.ejcb.2013.11.007 24461929

[B35] Kuleshov M. V. Jones M. R. Rouillard A. D. Fernandez N. F. Duan Q. Wang Z. (2016). Enrichr: A comprehensive gene set enrichment analysis web server 2016 update. Nucleic Acids Res. 44 (W1), W90–W97. 10.1093/nar/gkw377 27141961 PMC4987924

[B36] Lasham A. Print C. G. Woolley A. G. Dunn S. E. Braithwaite A. W. (2013). YB-1: Oncoprotein, prognostic marker, and therapeutic target? Biochem. J. 449 (1), 11–23. 10.1042/BJ20121323 23216250

[B37] Lee C. W. Zhan Q. Lezcano C. Frank M. H. Huang J. Larson A. R. (2014). Nestin depletion induces melanoma matrix metalloproteinases and invasion. Lab. Invest 94 (12), 1382–1395. 10.1038/labinvest.2014.130 25365206 PMC4419570

[B38] Liu H. Zhang Y. Li L. Cao J. Guo Y. Wu Y. (2021). Fascin actin-bundling protein 1 in human cancer: Promising biomarker or therapeutic target? Mol. Ther. - Oncolytics 20, 240–264. 10.1016/j.omto.2020.12.014 33614909 PMC7873579

[B39] Lu J. Li X. Wang F. Guo Y. Huang Y. Zhu H. (2017). YB-1 expression promotes pancreatic cancer metastasis that is inhibited by microRNA-216a. Exp. Cell Res. 359 (2), 319–326. 10.1016/j.yexcr.2017.07.039 28782557

[B40] Mordovkina D. Lyabin D. N. Smolin E. A. Sogorina E. M. Ovchinnikov L. P. Eliseeva I. (2020). Y-box binding proteins in mRNP assembly, translation, and stability control. Biomolecules 10 (4), 591. 10.3390/biom10040591 32290447 PMC7226217

[B41] Ostrowska-Podhorodecka Z. Ding I. Norouzi M. McCulloch C. A. (2022). Impact of vimentin on regulation of cell signaling and matrix remodeling. Front. Cell Dev. Biol. 10, 869069. 10.3389/fcell.2022.869069 35359446 PMC8961691

[B42] Panková K. Rösel D. Novotný M. Brábek J. (2010). The molecular mechanisms of transition between mesenchymal and amoeboid invasiveness in tumor cells. Cell Mol. Life Sci. 67 (1), 63–71. 10.1007/s00018-009-0132-1 19707854 PMC2801846

[B43] Sandru A. Voinea S. Panaitescu E. Blidaru A. (2014). Survival rates of patients with metastatic malignant melanoma. J. Med. Life 7 (4), 572–576. 25713625 PMC4316142

[B44] Sanz-Moreno V. Gadea G. Ahn J. Paterson H. Marra P. Pinner S. (2008). Rac activation and inactivation control plasticity of tumor cell movement. Cell 135 (3), 510–523. 10.1016/j.cell.2008.09.043 18984162

[B45] Satelli A. Li S. (2011). Vimentin in cancer and its potential as a molecular target for cancer therapy. Cell Mol. Life Sci. 68 (18), 3033–3046. 10.1007/s00018-011-0735-1 21637948 PMC3162105

[B46] Schittek B. Psenner K. Sauer B. Meier F. Iftner T. Garbe C. (2007). The increased expression of Y box-binding protein 1 in melanoma stimulates proliferation and tumor invasion, antagonizes apoptosis and enhances chemoresistance. Int. J. Cancer 120 (10), 2110–2118. 10.1002/ijc.22512 17266041

[B47] Schmitt M. Sinnberg T. Nalpas N. C. Maass A. Schittek B. Macek B. (2019). Quantitative proteomics links the intermediate filament nestin to resistance to targeted BRAF inhibition in melanoma cells. Mol. Cell Proteomics 18 (6), 1096–1109. 10.1074/mcp.RA119.001302 30890564 PMC6553926

[B48] Schwanhäusser B. Busse D. Li N. Dittmar G. Schuchhardt J. Wolf J. (2011). Global quantification of mammalian gene expression control. Nature 473 (7347), 337–342. 10.1038/nature10098 21593866

[B49] Shah J. V. Wang L. Z. Traub P. Janmey P. A. (1998). Interaction of vimentin with actin and phospholipids. Biol. Bull. 194 (3), 402–405. 10.2307/1543125 9664674

[B50] Shiota M. Izumi H. Onitsuka T. Miyamoto N. Kashiwagi E. Kidani A. (2008). Twist promotes tumor cell growth through YB-1 expression. Cancer Res. 68 (1), 98–105. 10.1158/0008-5472.CAN-07-2981 18172301

[B51] Sinnberg T. Sauer B. Holm P. Spangler B. Kuphal S. Bosserhoff A. (2012). MAPK and PI3K/AKT mediated YB-1 activation promotes melanoma cell proliferation which is counteracted by an autoregulatory loop. Exp. Dermatol. 21 (4), 265–270. 10.1111/j.1600-0625.2012.01448.x 22417301

[B52] Son H. Moon A. (2010). Epithelial-mesenchymal transition and cell invasion. Toxicol. Res. 26 (4), 245–252. 10.5487/TR.2010.26.4.245 24278531 PMC3834497

[B53] Sun B. O. Fang Y. Li Z. Chen Z. Xiang J. (2015). Role of cellular cytoskeleton in epithelial-mesenchymal transition process during cancer progression. Biomed. Rep. 3 (5), 603–610. 10.3892/br.2015.494 26405532 PMC4576489

[B54] Vig N. Rahman M. Gammon L. Peyric E. Mackenzie I. (2017). Investigation of the properties of the amoeboid cell, a new cell type in oral cancer. Lancet 389, S97. 10.1016/s0140-6736(17)30493-2

[B55] Wang N. Stamenović D. (2000). Contribution of intermediate filaments to cell stiffness, stiffening, and growth. Am. J. Physiol. Cell Physiol. 279 (1), C188–C194. 10.1152/ajpcell.2000.279.1.C188 10898730

[B56] Watanabe T. Kuramochi H. Takahashi A. Imai K. Katsuta N. Nakayama T. (2012). Higher cell stiffness indicating lower metastatic potential in B16 melanoma cell variants and in (-)-epigallocatechin gallate-treated cells. J. Cancer Res. Clin. Oncol. 138 (5), 859–866. 10.1007/s00432-012-1159-5 22297840 PMC11824207

[B57] Weder G. Hendriks-Balk M. C. Smajda R. Rimoldi D. Liley M. Heinzelmann H. (2014). Increased plasticity of the stiffness of melanoma cells correlates with their acquisition of metastatic properties. Nanomedicine 10 (1), 141–148. 10.1016/j.nano.2013.07.007 23891982

[B58] Werner A. C. Weckbach L. T. Salvermoser M. Pitter B. Cao J. Maier-Begandt D. (2020). Coronin 1B controls endothelial actin dynamics at cell-cell junctions and is required for endothelial network assembly. Front. Cell Dev. Biol. 8, 708. 10.3389/fcell.2020.00708 32850828 PMC7411154

[B59] Yamagishi A. Susaki M. Takano Y. Mizusawa M. Mishima M. Iijima M. (2019). The structural function of nestin in cell body softening is correlated with cancer cell metastasis. Int. J. Biol. Sci. 15 (7), 1546–1556. 10.7150/ijbs.33423 31337983 PMC6643143

[B60] Yu M. Bardia A. Wittner B. S. Stott S. L. Smas M. E. Ting D. T. (2013). Circulating breast tumor cells exhibit dynamic changes in epithelial and mesenchymal composition. science 339 (6119), 580–584. 10.1126/science.1228522 23372014 PMC3760262

