# Supplementary material for: Biomechanical and biochemical assessment of YB-1 expression in A375 melanoma cell line: Exploratory study
Source: Front Mol Med. 2023 Apr 20;3:1050487. doi: 10.3389/fmmed.2023.1050487 (PMC11285636; doi:10.3389/fmmed.2023.1050487)
Supplement: Supplementary file 1 [file Table1.DOCX]

Supplementary Material

**Supplementary Figure 1.** Cell stiffness as measured by the Young's modulus (kPa) is higher in the YB-1 expressing parental A375 cells when compared to the MelJuso cell line. Boxplots represent the difference in stiffness (kPa) measured by AFM between the two cell lines (A375 and MelJuso) (A), with the descriptive statistical analysis below (B). A significantly higher stiffness was observed in the A375 cell line compared to its corresponding MelJuso cell line (p < 0.001). *p < 0.05; Abbreviations: PA - parental; KO - knock-out; AFM - atomic force microscopy.

**Table 1**. Upregulated proteins in A375 knock-out cells. The information for the unregulated proteins presented in this table that do not have a citation were obtained by entering each protein name into [https://www.ncbi.nlm.nih.gov/protein/](https://atpscan.global.hornetsecurity.com/index.php?atp_str=uLtEy6QGGsmBd71BA5yow7nWm_z8hIYDvlJ562hLxEugWsV_lSsZLydlBsYbdvdL23f5wa2pp94xsa1dH80JyUQAf2XaXipYAzVF0kj9FDj-7WkdjBf9o00iq3faneTOXSxqFYElzFIimOjhlwUhdH_MyrIOiSYBklTXuNp5GB95EwSfIV_7q27xSaJZgQMgLvSw2dUcjINa8S9XjOdUKfnzTCuBgKDyA3fg2BS43QGKSYrKFEP528PCYGpR-dq7AO9OCAG6F_0yzeINJLW3pwxZTLqN7qdivF5B54RlwAKfo_lGYZhqcdvWN6KZ2UfrPs_g3BM2o2qVdcaquiM6OiM5XEddC2UA5JSweCEjOjojcvoAfUqGtC29kgDD7oPBdw). The data is freely available.

| Gene Name | Protein Name | Effect size (fold change) | Functional information |
| --- | --- | --- | --- |
| TGM2 | Protein-glutamine gamma-glutamyltransferase 2 | -5.82 | - Catalyzes the cross-linking of proteins and the conjugation of polyamines to proteins.  - Activates NF-κB signaling phatway, which leads to the upregulation of CD44 and promotes EMT phenotype and promotes cancer stem cell survival through the Wnt/β-catenin pathway [1]. |
| SFN | 14-3-3 protein sigma | -5.04 | - Adapter protein.  - Regulates protein synthesis and epithelial cell growth by stimulating Akt/mTOR pathway and is also involved in the p53/TP53 signaling [2].  - Silencing of 14-3-3σ in melanoma metastases might contribute to tumor progression [3]. |
| LIMA1 | LIM domain and actin-binding protein 1 | -4.18 | - Increases the number and size of actin stress fibers and inhibits membrane ruffling and actin filament depolymerization [4].  - Acts as a mechanotransmitter, allowing vinculin binding to α-catenin and formation of a secondary molecular bond between the adherens complex and the cytoskeleton through vinculin [5]. |
| ZYX | Zyxin | -3.97 | - Adhesion plaque protein.  - Binds alpha-actinin and the CRP protein.  - Important for targeting TES and ENA/VASP family members to focal adhesions and for the formation of actin-rich structures [6].  - High ZYX expression was associated with worse prognosis and the ZYX-STMN1 axis might be a potential therapeutic target for glioblastoma [7]. |
| EHD2 | EH domain-containing protein 2 | -3.92 | - ATP- and membrane-binding protein, controls membrane reorganization/tubulation upon ATP hydrolysis.  - Down-regulation of EHD2 has been associated with migration and invasion by abrogating the expression of Rac1 and regulating the epithelial-mesenchymal transition (EMT) key markers E-cadherin in breast cancer [8]. |
| CAPG | Macrophage-capping protein | -3.79 | - Calcium-sensitive protein which reversibly blocks the barbed ends of actin filaments but does not sever preformed actin filaments.  - May play a role in regulating cytoplasmic and/or nuclear structures through potential interactions with actin.  - Gelsolin/villins. mediator of cross-talk between the actin cytoskeleton and microtubule-based organelles that regulate cell division [9, 10]. |
| RAP1B | Ras-related protein Rap-1b | -3.48 | - GTP-binding protein with GTPase activity. - Altered RAP1B expression occurs in several types of human cancer and that RAP1B promotes cancer growth, invasion and metastasis [11, 12]. |
| RPS29 | Small subunit ribosomal protein s29e | -3.31 | - Has endonuclease activity and induces apoptosis in on-small cell lung cancer cells and sensitizes them to chemotherapy [13]. |
| TMOD3 | Tropomodulin-3 | -3.29 | - Blocks the elongation and depolymerization of the actin filaments at the pointed end. - The Tmod/TM complex contributes to the formation of the short actin protofilament [14]. |
| FAM120A | Constitutive coactivator of PPAR-gamma-like protein 1 | -3.17 | - Required for the activation and recruitment of FAK, Src, PI3K, and most of the proteins involved in IL13Rα2 signaling. It also activates src family kinases and acts as a scaffolding protein enabling src family kinases to phosphorylate and activate PI3-kinase [15]. |
| PDLIM5 | PDZ and LIM domain protein 5 | -3.14 | - Cytoskeleton related protein and may play an important role in the invasion and metastasis [16]. |
| EEA1 | Early endosome antigen 1 | -3.02 | - Binds phospholipid vesicles containing phosphatidylinositol 3-phosphate and participates in endosomal trafficking [17]. - Zinc fingers FYVE-type [18] / VEGF signaling pathway [19]. |
| TUBB6 | Tubulin beta-6 chain | -2.88 | - Tubulin is the major constituent of microtubules.  - TUBB6 is used as a prognostic biomarker in many cancers entities [20]. |
| CFL2 | Cofilin-2 | -2.88 | - Controls reversibly actin polymerization and depolymerization.  - Binds to G- and F-actin.  - Promotes the cell motility by regulating the cytoskeletal reorganization, promoting the lamellipodium formation, cell–cell adhesion and epithelial-to-mesenchymal transition (EMT) process [21]. |
| AKAP12 | A-kinase anchor protein 12 | -2.84 | - Mediates the subcellular compartmentation of protein kinase A (PKA) and protein kinase C (PKC).  - Functions as a metastasis suppressor through its ability to scaffold multiple mediators of oncogenic signaling, including PKC, PKA, Src-family kinases (SFK), phosphoinositol phosphates, F-actin and cyclins [22, 23]. |
| PAK2 | Serine/threonine-protein kinase PAK 2 | -2.62 | - Plays a role in a variety of different signaling pathways including cytoskeleton regulation, cell motility, cell cycle progression, apoptosis or proliferation [24].  - Acts as downstream effector of the small GTPases CDC42 and RAC1. Activation by the binding of active CDC42 and RAC1 results in a conformational change and a subsequent autophosphorylation on several serine and/or threonine residues [25].  - Stimulates cell survival and cell growth. |
| SEPTIN11 | Septin-11 | -2.55 | - Filament-forming cytoskeletal GTPase.  - Possible role in cytokinesis [26].  - May play a role in the cytoarchitecture of neurons, including dendritic arborization and dendritic spines, and in GABAergic synaptic connectivity [27]. |
| TJP1 | Tight junction protein ZO-1 | -2.54 | - Involved in transducing a signal required for tight junction assembly, while the C-terminal may have specific properties of tight junctions [28]. – Regulates cell migration by targeting CDC42BPB to the leading edge of migrating cells [29]. |
| SDCBP | Syntenin-1 | -2.48 | - Involved in a variety of functions such as: trafficking of transmembrane proteins, neuro and immunomodulation, exosome biogenesis, and tumorigenesis.  - Positively regulates TGFB1- mediated SMAD2/3 activation and TGFB1-induced epithelial-to- mesenchymal transition (EMT) and cell migration [30]. |
| RCN1 | Reticulocalbin-1 | -2.48 | - Regulates calcium-dependent activities with a strict expression in the endoplasmic reticulum [31]. |
| PTPN1 | Tyrosine-protein phosphatase non-receptor type 1 | -2.40 | - Acts as a regulator of endoplasmic reticulum unfolded protein response.  - Mediates dephosphorylation of EIF2AK3/PERK; inactivating the protein kinase activity of EIF2AK3/PERK.  - Involved in the hepatocyte growth factor receptor signaling pathway through dephosphorylation of ME T [32]. |
| CAST | Calpastatin | -2.31 | - Specific inhibition of calpain (a calcium dependent cysteine protease) [33]. |
| CAPN1 | Calpain-1 catalytic subunit | -2.15 | - Calcium-regulated non-lysosomal thiol-protease which catalyzes limited proteolysis of substrates involved in cytoskeletal remodeling and signal transduction [34]. |
| DCTN1 | Dynactin subunit 1 | -2.15 | - Plays a key role in dynein-mediated retrograde transport of vesicles and organelles along microtubules by recruiting and tethering dynein to microtubules. Binds to both dynein and microtubules providing a link between specific cargos, microtubules and dynein [35] . |
| PRKACB | cAMP-dependent protein kinase catalytic subunit beta | -2.11 | - Regulates diverse cellular processes such as cell proliferation, the cell cycle, differentiation and regulation of microtubule dynamics, chromatin condensation and decondensation, nuclear envelope disassembly and reassembly, as well as regulation of intracellular transport mechanisms. |
| EGFR | Epidermal growth factor receptor | -2.09 | - Receptor tyrosine kinase binding ligands of the EGF family and activating several signaling cascades to convert extracellular cues into appropriate cellular responses. |
| PPME1 | Protein phosphatase methylesterase 1 | -2.08 | - Demethylates proteins that have been reversibly carboxymethylated. Demethylates PPP2CB (in vitro) and PPP2CA. Binding to PPP2CA displaces the manganese ion and inactivates the enzyme. |
| ARPC3 | Actin-related protein 2/3 complex subunit 3 | -2.04 | - Functions as component of the Arp2/3 complex which is involved in regulation of actin polymerization and together with an activating nucleation-promoting factor mediates the formation of branched actin networks. |
| CORO1B | Coronin-1B | -1.95 | - Regulates leading edge dynamics and cell motility in fibroblasts. Possible involvement in cytokinesis and signal transduction. |
| SNX1 | Sorting nexin-1 | -1.68 | - Involved in several stages of intracellular trafficking. Interacts with membranes containing phosphatidylinositol 3- phosphate (PtdIns(3P)) or phosphatidylinositol 3,5-bisphosphate (PtdIns(3,5)P2).  - Acts in part as component of the retromer membrane-deforming SNX-BAR subcomplex. |
| DBNL | Drebrin-like protein | -1.67 | - Adapter protein that binds F-actin and DNM1, and plays a role in receptor-mediated endocytosis.  - Involved in the reorganization of the actin cytoskeleton, formation of cell projections, such as neurites, in neuron morphogenesis and synapse formation via its interaction with WASL and COBL. Does not bind G- actin and promote actin polymerization by itself. Required for the formation of organized podosome rosettes. |
| FLII | Flii, actin remodeling protein | -1.62 | - Protein flightless-1 homolog.  - Role as coactivator in transcriptional activation by hormone-activated nuclear receptors (NR) and acts in cooperation with NCOA2 and CARM1.  - May play a role in regulation of cytoskeletal rearrangements involved in cytokinesis and cell migration, by inhibiting Rac1-dependent paxillin phosphorylation [36]. |
| FHL1 | Four and a half LIM domains protein 1 | -1.60 | - Involvement in muscle development or hypertrophy; LIM domain containing. |
| ACOT9 | Acyl-coenzyme A thioesterase 9, mitochondrial | -1.59 | - Acyl-CoA thioesterases are a group of enzymes that catalyze the hydrolysis of acyl-CoAs to the free fatty acid and coenzyme A (CoASH), providing the potential to regulate intracellular levels of acyl-CoAs, free fatty acids and CoASH. |
| NEK7 | Nima (never in mitosis gene a)-related kinase 7 | -1.58 | - Serine/threonine-protein kinase Nek7;  - Plays an important role in mitotic cell cycle progression. Required for microtubule nucleation activity of the centrosome, robust mitotic spindle formation and cytokinesis. |
| FERMT2 | Fermitin family homolog 2 | -1.53 | - Scaffolding protein that enhances integrin activation mediated by TLN1 and/or TLN2, but activates integrins only weakly by itself. Binds to membranes enriched in phosphoinositides.  - Enhances integrin-mediated cell adhesion onto the extracellular matrix and cell spreading; this requires both its ability to interact with integrins and with phospholipid membranes.  - Required for the assembly of focal adhesions.  - Participates in the connection between extracellular matrix adhesion sites and the actin cytoskeleton and also in the orchestration of actin assembly. |
| MAP1LC3B | Microtubule-associated proteins 1A/1B light chain 3B | -1.47 | - Ubiquitin-like modifier involved in formation of autophagosomal vacuoles (autophagosomes).  - Plays a role in mitophagy which contributes to regulate mitochondrial quantity and quality by eliminating the mitochondria to a basal level to fulfill cellular energy requirements and preventing excess ROS production. |
| CAV1 | Caveolin-1 | -1.47 | - May act as a scaffolding protein within caveolar membranes. Interacts directly with G-protein alpha subunits and can functionally regulate their activity.  - Involved in the costimulatory signal essential for T-cell receptor (TCR)- mediated T-cell activation. Its binding to DPP4 induces T-cell proliferation and NF-kappa-B activation in a T-cell receptor/CD3- dependent manner.  - CAV1 promotes cancer cell progression and metastasis through Wnt/β-catenin pathway [37]. |
| ITGA2 | Integrin alpha-2 | -1.46 | -Integrin alpha-2/beta-1 is a receptor for laminin, collagen, collagen C-propeptides, fibronectin and E-cadherin. It recognizes the proline-hydroxylated sequence G-F-P-G-E-R in collagen I.  - Responsible for adhesion of platelets and other cells to collagens, modulation of collagen and collagenase gene expression, force generation and organization of newly synthesized extracellular matrix. |
| VCL | Vinculin | -1.44 | - Actin filament (F-actin)-binding protein involved in cell-matrix adhesion and cell-cell adhesion.  - Regulates cell- surface E-cadherin expression and potentiates mechanosensing by the E-cadherin complex.  - May also play important roles in cell morphology and locomotion. |
| PBK | Lymphokine-activated killer T-cell-originated protein kinase | -1.39 | - Phosphorylates MAP kinase p38 and is active only in mitosis.  - When phosphorylated, forms a complex with TP53, leading to TP53 destabilization and attenuation of G2/M checkpoint during doxorubicin-induced DNA damage; Belongs to the protein kinase superfamily. |
| CEP170 | Centrosomal protein of 170 kDa | -1.38 | - Plays a role in microtubule organization. Required for centriole subdistal appendage assembly; Belongs to the CEP170 family. |
| DBN1 | Drebrin 1 | -1.38 | - Drebrins might play some role in cell migration, extension of neuronal processes and plasticity of dendrites. Required for actin polymerization at immunological synapses (IS). |
| GMFB | Glia maturation factor beta | -1.38 | - Causes differentiation of brain cells, stimulation of neural regeneration, and inhibition of proliferation of tumor cells [38]. |
| FLNB | Filamin-B | -1.36 | - Connects cell membrane constituents to the actin cytoskeleton. May promote orthogonal branching of actin filaments and links actin filaments to membrane glycoproteins.  - Anchors various transmembrane proteins to the actin cytoskeleton.  - Various interactions and localizations of isoforms affect myotube morphology and myogenesis. |
| PP4C | Serine/threonine-protein phosphatase 4 catalytic subunit | -1.32 | - Involved in many processes such as microtubule organization at centrosomes, maturation of spliceosomal snRNPs, apoptosis, DNA repair, tumor necrosis factor (TNF)-alpha signaling, activation of c-Jun N-terminal kinase MAPK8, regulation of histone acetylation, DNA damage checkpoint signaling, NF-kappa-B activation and cell migration. The PPP4C- PPP4R1 PP4 complex may play a role in dephosphorylation and regulation of HDAC3. |
| SEPTIN10 | Septin-10 | -1.27 | - Filament-forming cytoskeletal GTPase. |
| LMO7 | LIM domain only protein 7 | -1.27 | - Roles in embryonic development and breast cancer progression [39]. |
| ZO2 | Tight junction protein ZO-2 | -1.27 | - Plays a role in tight junctions and adherens junctions. |
| AHNAK | Neuroblast differentiation-associated protein AHNAK | -1.25 | - May be required for neuronal cell differentiation [40]. |
| VAPA | Vesicle-associated membrane protein-associated protein A | -1.25 | - Present in the plasma membrane and intracellular vesicles and may also be associated with the cytoskeleton and may function in vesicle trafficking, membrane fusion, protein complex assembly and cell motility. |
| SLC9A3R2 | Na(+)/H(+) exchange regulatory cofactor NHE-RF2 | -1.16 | - Scaffold protein that connects plasma membrane proteins with members of the ezrin/moesin/radixin family and thereby helps to link them to the actin cytoskeleton and to regulate their surface expression. |
| EZR | Villin 2 (ezrin) | -1.14 | - Involved in connections of major cytoskeletal structures to the plasma membrane. In epithelial cells, required for the formation of microvilli and membrane ruffles on the apical pole. |
| CSRP1 | Cysteine and glycine-rich protein 1 | -1.13 | - Could play a role in neuronal development [41]; LIM domain containing. |
| CORO1A | Coronin-1A | -1.11 | - May be a crucial component of the cytoskeleton of highly motile cells, functioning both in the invagination of large pieces of plasma membrane, as well as in forming protrusions of the plasma membrane involved in cell locomotion. |
| FLNA | Filamin-A | -1.11 | - Promotes orthogonal branching of actin filaments and links actin filaments to membrane glycoproteins. Plays a role in cell-cell contacts and adherens junctions. |
| STAT3 | Signal transducer and activator of transcription 3 | -1.09 | - Signal transducer and transcription activator that mediates cellular responses to interleukins and various growth factors. - Binds to the interleukin-6 (IL-6)-responsive elements identified in the promoters of various acute-phase protein genes [42]. |
| CORO1C | Coronin-1C | -1.06 | - May be involved in cytokinesis, motility, and signal transduction. |
| RAE1 | mRNA export factor | -1.03 | - Plays a role in mitotic bipolar spindle formation. Binds mRNA. May function in nucleocytoplasmic transport and in directly or indirectly attaching cytoplasmic mRNPs to the cytoskeleton. |
| NSUN2 | NOP2/Sun RNA methyltransferase family member 2 | -1.02 | - tRNA (cytosine(34)-C(5))-methyltransferase; May act downstream of Myc to regulate epidermal cell growth and proliferation. |
| PTPLAD1 | Very-long-chain (3R)-3-hydroxyacyl-CoA dehydratase 3 | -1.02 | - Catalyzes the third of the four reactions of the long- chain fatty acids elongation cycle. Overexpressed in >90% of studied primary melanoma lesions [43]. |
| KTN1 | Kinectin | -1.01 | - Receptor for kinesin thus involved in kinesin-driven vesicle motility.  - Accumulates in integrin-based adhesion complexes (IAC) upon integrin aggregation by fibronectin. |

**Table 2**.  Upregulated proteins in A375 parental cells. The information for the unregulated proteins presented in this table that do not have a citation were obtained by entering each protein name into [https://www.ncbi.nlm.nih.gov/protein/](https://atpscan.global.hornetsecurity.com/index.php?atp_str=K7Wqo0LYX7Xeh_HsHlJZQCBwRyo9k-5J7Ty2q-IwtfM4AkY_kEa9UgZXunQ8ykFF7U_yHTrtkKTw1j5-ajbswzZRuK8s8jCJD0WTxi3Sca4Ng3bDyI6MDRziHTfSzWztO6SbKwBJRKDZvVCHoYBX9OzxJi8MgAsLFvJdwEHyKqfOVcK7IE9M4Rt-vAhqxNgxUlHa6CxTcwPWfcW58j6qb3wWwmIXRMsX8rTHbFLFK_4mdCi_7v0aNLQ6PcJuiXCBArV1OaONKO-87-3P9PPUoV5owPdyzRxgo-45m6UFXBzJTdZyV_Zno7pwOkPJvyAsLxmEVzRcsgdVpmDz4iM6OiM9PKqTYS22VIAkW-0jOjoj4TE9h9jcPvsJr7sAwc93gg). The data is freely available.

| Gene Name | Protein Name | Effect size (fold change) | Functional information |
| --- | --- | --- | --- |
| NES | Nestin | 6.73 | - In cutaneous melanoma, nestin has been reported to be increasingly expressed in more advanced stages [44, 45]  -Considered as a marker of malignancy of neuroectodermal origin including melanoma, where it was recognized as a prognostic factor [46].  - Plays a role in connecting components of the cytoskeleton as it copolymerizes into filaments with class III intermediate filament proteins, mostly vimentin, contributing to its disassembly[47]. Also, expression of YB-1 was also associated with significant preferential expression of vimentin, which is widely expressed in mesenchymal cells, and which has been recognized as a marker of EMT where immotile cells convert to motile mesenchymal cells [48]. |
| YBX1 | Y-box binding protein 1 | 4.68 | - Is an oncogenic cold shock domain family member that can act both as a transcription factor in the nucleus and as a translational regulator in the cytoplasm. - It overexpressed in malignant melanoma with increasing protein levels during tumor progression and is associated with poor prognosis strongly suggests an important role of YB-1 in melanoma development and progression [49, 50].  - Is a component of exosomes involved in sorting and packaging of mRNAs and miRNAs / important packaging protein for mRNAs in the cytoplasm, enhancing their stability and regulating their translational activity [51, 52].  - It is a part of messenger ribonucleoprotein particles (mRNPs) and controls translation and stability of mRNA in a dose-dependent manner [50]. |
| SUPT16H | FACT complex subunit SPT16 | 4.34 | - Is a transcription elongation factor that comprises the structure-specific recognition protein 1 (SSRP1) and the protein SUPT16H subunits.  - Both FACT subunits, SSRP1 and SPT16, are significantly elevated in breast cancer samples *versus* normal mammary epithelial cells [53] and the level of FACT system defines the transcriptional scenery and aggressive phenotype of breast cancer cells [54]. |
| BOP1 | Ribosome biogenesis protein BOP1 | 3.81 | - Is a conserved RNA-binding protein involved in ribosome biogenesis, cell cycle, and cell proliferation [55] and is part of the trimeric “PeBoW” complex, essential for ribosome biogenesis and cell cycle progression [56].  - In melanoma cell lines, high BOP1 expression caused an increase in proliferation and colony formation, whereas low BOP1 expression conferred reduced proliferation, colony formation, and a senescent like phenotype [57].  - Is a conserved RNA-binding protein involved in ribosome biogenesis, cell cycle, and cell proliferation [55].  - Knockdown of BOP1 attenuates vascular smooth muscle cell proliferation and migration via activating the p53-dependent pathway [58].  - Is dysregulated in several cancers and is involved in promoting the tumor occurrence and progression [59]. |
| SNRPE | Small nuclear ribonucleoprotein E | 3.62 | - Core component of the spliceosomal U1, U2, U4 and U5 small nuclear ribonucleoproteins (snRNPs).  - SNRPE knockdown leads to mTOR downregulation, targeting SNRPE or SNRPD1 leads to cell death through autophagy [60]. |
| MRT4 | MRT4 homolog, ribosome maturation factor | 3.23 | - Component of the ribosome assembly machinery. |
| WDR12 | Ribosome biogenesis protein WDR12 | 2.78 | - Component of the PeBoW complex, which is required for maturation of 28S and 5.8S ribosomal RNAs and formation of the 60S ribosome. WDR12 functions together with Pes1 and Bop1 to promote the progression of the cell cycle [56]. - Suppression of Ribosome Biogenesis by Targeting WD Repeat Domain 12 (WDR12) Inhibits Glioma Stem-Like Cell Growth [61]. |
| FSCN1 | Fascin-1 | 2.64 | - Is a filamentous actin-binding protein that crosslinks actin microfilaments into tight bundles. These are important for the formation of microspikes, filopodia, and invadopodia and their functionality in cell migration, cell-matrix adhesion, and cell-to-cell interactions.  - Can serve as a reliable immunohistochemical marker in distinguishing malignant melanomas from melanocytic nevi and dysplastic nevi [62].  - Fascin-1 expression does not correlate with progression in melanoma [63]. |
| DHX30 | Putative ATP-dependent RNA helicase DHX30 | 2.62 | - Plays an important role in the assembly of the mitochondrial large ribosomal subunit and contributes to cancer cell survival [64]. |
| DDX27 | Probable ATP-dependent RNA helicase DDX27 | 2.56 | - Component of the nucleolar ribosomal RNA (rRNA) processing machinery that regulates 3' end formation of ribosomal 47S rRNA. DEAD-box helicase 27 enhances stem cell-like properties with poor prognosis in breast cancer [65]. |
| NIP7 | 60S ribosome subunit biogenesis protein NIP7 homolog | 2.47 | - Required for proper 34S pre-rRNA processing and 60S ribosome subunit assembly. |
| LSM6 | U6 snRNA-associated Sm-like protein LSm6 | 2.34 | - Component of LSM protein complexes, which are involved in RNA processing Component of the cytoplasmic LSM1-LSM7 complex, which is thought to be involved in mRNA degradation by activating the decapping step in the 5'-to-3' mRNA decay pathway.  - Highly expressed in the cisplatin resistant melanoma cell line M24met and downregulated upon cisplatin treatment [66] . |
| Q5JRI1 | Fus-interacting serine-arginine-rich protein 1 | 2.32 | - Splicing factor that in its dephosphorylated form acts as a general repressor of pre-mRNA splicing. |
| SEPT9 |  | 2.26 | - Only member of the septin family that directly promotes actin polymerization and cross-linking.  - It is involved in SEPT9-dependent F-actin cross-linking which enables the generation of F-actin bundles required for the sustained stabilization of highly contractile actomyosin structures [67]. |
| NOB1 | RNA-binding protein NOB1 | 2.26 | - Plays a role in mRNA degradation. |
| NOG1 | Nucleolar GTP-binding protein 1 | 2.13 | - Involved in the biogenesis of the 60S ribosomal subunit. |
| EXOSC4 | Exosome complex component RRP41 | 2.10 | - Non-catalytic component of the RNA exosome complex that participates in a multitude of cellular RNA processing and degradation events. |
| SF3B5 | Splicing factor 3B subunit 5 | 2.09 | - Involved in pre-mRNA splicing as a component of the splicing factor SF3B complex. |
| VIM | Vimentin | 2.08 | - Is a member of intermediate filaments, characteristic of mesenchymal cells, major structural components of the cytoskeleton responsible contraction and migration of cells.  - Vimentin and actin associate with integrins and where vinculin and plectin recruited were termed as the vimentin associated matrix adhesions (VAMs) [68].  - Used as a marker to diagnose human melanoma clinically,  and is significantly associated with melanoma hematogenous metastasis [69]. |
| RRP9 | U3 small nucleolar RNA-interacting protein 2 | 2.08 | - Component of a nucleolar small nuclear ribonucleoprotein particle (snoRNP) thought to participate in the processing and modification of pre-ribosomal RNA |
| NOG2 | Nucleolar GTP-binding protein 2 | 2.05 | - GTPase that associates with pre-60S ribosomal subunits in the nucleolus and is required for their nuclear export and maturation. |
| WDR3 | WD repeat-containing protein 3 | 2.04 | - Belongs to the WD repeat WDR3/UTP12 family. |
| RRS1 | Ribosome biogenesis regulatory protein homolog | 2.03 | - Involved in ribosomal large subunit assembly. |
| DDX54 | ATP-dependent RNA helicase DDX54 | 1.96 | - Represses the transcriptional activity of nuclear receptors. |
| RRP15 | RRP15-like protein | 1.94 | - Ribosomal RNA processing 15 homolog. |
| SNRPB2 | U2 small nuclear ribonucleoprotein B | 1.92 | - Involved in pre-mRNA splicing. |
| NOP16 | NOP16 nucleolar protein | 1.92 | - Belongs to the NOP16 family. |
| MAK16 | Protein MAK16 homolog | 1.88 | - RNA binding motif containing. |
| WDR36 | WD repeat-containing protein 36 | 1.84 | - Involved in the nucleolar processing of SSU 18S rRNA.  - Involved in T-cell activation and highly coregulated with IL2. |
| NOL11 | Nucleolar protein 11 | 1.73 | - Ribosome biogenesis factor. |
| DDX56 | Probable ATP-dependent RNA helicase DDX56 | 1.70 | - May play a role in later stages of the processing of the pre-ribosomal particles leading to mature 60S ribosomal subunits. |
| SRSF5 | Splicing factor, arginine/serine-rich 4/5/6 | 1.69 | - Serine/arginine-rich splicing factor 5. |
| SSRP1 | Structure-specific recognition protein 1 | 1.51 | - FACT complex subunit SSRP1; replicates, transcribes, and repairs DNA. - elevated SSRP1 expression has been linked to metastasized tumors, making SSRP1 a potential prognostic marker and an anticancer target for tumor inhibition [70, 71]. |
| EXOSC7 | Exosome complex component RRP42 | 1.48 | - Non-catalytic component of the RNA exosome complex which has 3'->5' exoribonuclease activity and participates in a multitude of cellular RNA processing and degradation events. |
| PPAN | Ribosome biogenesis protein ssf1/2 | 1.42 | - Suppressor of SWI4 1 homolog; May have a role in cell growth. |
| ADAR | Double-stranded RNA-specific adenosine deaminase | 1.14 | - Catalyzes the hydrolytic deamination of adenosine to inosine in double-stranded RNA (dsRNA) referred to as A-to-I RNA editing. |
| WDR43 | WD repeat-containing protein 43 | 1.13 | - Ribosome biogenesis factor. |
| SPB1 | Putative tRNA (cytidine(32)/guanosine(34)-2'-O)-methyltransferase | 1.13 | - Methylates the 2'-O-ribose of nucleotides at positions 32 and 34 of the tRNA anticodon loop of substrate tRNAs. |
| NOL10 | Nucleolar protein 10 | 1.10 | - WD repeat domain containing; |
| SUPT5H | Transcription elongation factor SPT5 | 1.10 | - Component of the DRB sensitivity-inducing factor complex (DSIF complex), which regulates mRNA processing and transcription elongation by RNA -polymerase II. |
| QKI | Qki, kh domain containing rna binding | 1.04 | - RNA-binding protein that plays a central role in myelinization. |
| PABP4 | Polyadenylate-binding protein 4 | 1.02 | - Binds the poly(A) tail of mRNA. |
| HNRPC | Heterogeneous nuclear ribonucleoproteins C1/C2 | 1.01 | - Binds pre-mRNA and nucleates the assembly of 40S hnRNP particles. |

**References**

1. Condello, S.; Sima, L.; Ivan, C.; Cardenas, H.; Schiltz, G.; Mishra, R. K.; Matei, D., Tissue Tranglutaminase Regulates Interactions between Ovarian Cancer Stem Cells and the Tumor NicheTissue Transglutaminase in Ovarian Cancer Stem Cells. *Cancer research* **2018,** 78, (11), 2990-3001.

2. Yang, H. Y.; Wen, Y. Y.; Chen, C. H.; Lozano, G.; Lee, M. H., 14-3-3 sigma positively regulates p53 and suppresses tumor growth. *Mol Cell Biol* **2003,** 23, (20), 7096-107.

3. Schultz, J.; Ibrahim, S. M.; Vera, J.; Kunz, M., 14-3-3sigma gene silencing during melanoma progression and its role in cell cycle control and cellular senescence. *Mol Cancer* **2009,** 8, 53.

4. Maul, R. S.; Song, Y.; Amann, K. J.; Gerbin, S. C.; Pollard, T. D.; Chang, D. D., EPLIN regulates actin dynamics by cross-linking and stabilizing filaments. *J Cell Biol* **2003,** 160, (3), 399-407.

5. Chervin-Pétinot, A.; Courçon, M.; Almagro, S.; Nicolas, A.; Grichine, A.; Grunwald, D.; Prandini, M. H.; Huber, P.; Gulino-Debrac, D., Epithelial protein lost in neoplasm (EPLIN) interacts with α-catenin and actin filaments in endothelial cells and stabilizes vascular capillary network in vitro. *J Biol Chem* **2012,** 287, (10), 7556-72.

6. Oldenburg, J.; van der Krogt, G.; Twiss, F.; Bongaarts, A.; Habani, Y.; Slotman, J. A.; Houtsmuller, A.; Huveneers, S.; de Rooij, J., VASP, zyxin and TES are tension-dependent members of Focal Adherens Junctions independent of the α-catenin-vinculin module. *Sci Rep* **2015,** 5, 17225.

7. Wen, X.-M.; Luo, T.; Jiang, Y.; Wang, L.-H.; Luo, Y.; Chen, Q.; Yang, K.; Yuan, Y.; Luo, C.; Zhang, X.; Yan, Z.-X.; Fu, W.-J.; Tan, Y.-H.; Niu, Q.; Xiao, J.-F.; Chen, L.; Wang, J.; Huang, J.-F.; Cui, Y.-H.; Zhang, X.; Wang, Y.; Bian, X.-W., Zyxin (ZYX) promotes invasion and acts as a biomarker for aggressive phenotypes of human glioblastoma multiforme. *Laboratory Investigation* **2020,** 100, (6), 812-823.

8. Yang, X.; Ren, H.; Yao, L.; Chen, X.; He, A., Role of EHD2 in migration and invasion of human breast cancer cells. *Tumor Biology* **2015,** 36, (5), 3717-3726.

9. Jiang, S.; Yang, Y.; Zhang, Y.; Ye, Q.; Song, J.; Zheng, M.; Li, X., Overexpression of CAPG Is Associated with Poor Prognosis and Immunosuppressive Cell Infiltration in Ovarian Cancer. *Dis Markers* **2022,** 2022, 9719671.

10. Hubert, T.; Van Impe, K.; Vandekerckhove, J.; Gettemans, J., The F-actin filament capping protein CapG is a bona fide nucleolar protein. *Biochem Biophys Res Commun* **2008,** 377, (2), 699-704.

11. Cui, G.; Wang, C.; Lin, Z.; Feng, X.; Wei, M.; Miao, Z.; Sun, Z.; Wei, F., Prognostic and immunological role of Ras-related protein Rap1b in pan-cancer. *Bioengineered* **2021,** 12, (1), 4828-4840.

12. Xu, J. H.; Zhao, W. Y.; Fang, Q. Q.; Wang, X. F.; Zhang, D. D.; Hu, Y. Y.; Zheng, B.; Tan, W. Q., Long Noncoding RNA LUADT1 Is Upregulated in Melanoma and May Sponge miR-28-5p to Upregulate RAP1B. *Cancer Biother Radiopharm* **2020,** 35, (4), 307-312.

13. Khanna, N.; Sen, S.; Sharma, H.; Singh, N., S29 ribosomal protein induces apoptosis in H520 cells and sensitizes them to chemotherapy. *Biochem Biophys Res Commun* **2003,** 304, (1), 26-35.

14. Chu, X.; Thompson, D.; Yee, L. J.; Sung, L. A., Genomic organization of mouse and human erythrocyte tropomodulin genes encoding the pointed end capping protein for the actin filaments. *Gene* **2000,** 256, (1-2), 271-81.

15. Bartolomé, R. A.; García-Palmero, I.; Torres, S.; López-Lucendo, M.; Balyasnikova, I. V.; Casal, J. I., IL13 Receptor α2 Signaling Requires a Scaffold Protein, FAM120A, to Activate the FAK and PI3K Pathways in Colon Cancer MetastasisIL13Rα2 Signaling in Colorectal Cancer Metastasis. *Cancer research* **2015,** 75, (12), 2434-2444.

16. Liu, X.; Chen, L.; Huang, H.; Lv, J. M.; Chen, M.; Qu, F. J.; Pan, X. W.; Li, L.; Yin, L.; Cui, X. G.; Gao, Y.; Xu, D. F., High expression of PDLIM5 facilitates cell tumorigenesis and migration by maintaining AMPK activation in prostate cancer. *Oncotarget* **2017,** 8, (58), 98117-98134.

17. Kutateladze, T. G., Phosphatidylinositol 3-phosphate recognition and membrane docking by the FYVE domain. *Biochim Biophys Acta* **2006,** 1761, (8), 868-77.

18. Gaullier, J. M.; Ronning, E.; Gillooly, D. J.; Stenmark, H., Interaction of the EEA1 FYVE finger with phosphatidylinositol 3-phosphate and early endosomes. Role of conserved residues. *J Biol Chem* **2000,** 275, (32), 24595-600.

19. E, G.; Cao, Y.; Bhattacharya, S.; Dutta, S.; Wang, E.; Mukhopadhyay, D., Endogenous Vascular Endothelial Growth Factor-A (VEGF-A) Maintains Endothelial Cell Homeostasis by Regulating VEGF Receptor-2 Transcription*. *Journal of Biological Chemistry* **2012,** 287, (5), 3029-3041.

20. Gabbireddy, S. R.; Vosatka, K. W.; Chung, A. J.; Logue, J. S., Melanoma cells adopt features of both mesenchymal and amoeboid migration within confining channels. *Sci. Rep.* **2021,** 11, (1), 17804.

21. Xu, J.; Huang, Y.; Zhao, J.; Wu, L.; Qi, Q.; Liu, Y.; Li, G.; Li, J.; Liu, H.; Wu, H., Cofilin: A Promising Protein Implicated in Cancer Metastasis and Apoptosis. *Front Cell Dev Biol* **2021,** 9, 599065.

22. Gelman, I. H., Suppression of tumor and metastasis progression through the scaffolding functions of SSeCKS/Gravin/AKAP12. *Cancer Metastasis Rev* **2012,** 31, (3-4), 493-500.

23. Muramatsu, M.; Akakura, S.; Gao, L.; Peresie, J.; Balderman, B.; Gelman, I. H., SSeCKS/Akap12 suppresses metastatic melanoma lung colonization by attenuating Src-mediated pre-metastatic niche crosstalk. *Oncotarget* **2018,** 9, (71), 33515-33527.

24. Dummler, B.; Ohshiro, K.; Kumar, R.; Field, J., Pak protein kinases and their role in cancer. *Cancer Metastasis Rev* **2009,** 28, (1-2), 51-63.

25. Jung, J. H.; Traugh, J. A., Regulation of the interaction of Pak2 with Cdc42 via autophosphorylation of serine 141. *J Biol Chem* **2005,** 280, (48), 40025-31.

26. Estey, M. P.; Di Ciano-Oliveira, C.; Froese, C. D.; Bejide, M. T.; Trimble, W. S., Distinct roles of septins in cytokinesis: SEPT9 mediates midbody abscission. *J Cell Biol* **2010,** 191, (4), 741-9.

27. Li, X.; Serwanski, D. R.; Miralles, C. P.; Nagata, K. I.; De Blas, A. L., Septin 11 is present in GABAergic synapses and plays a functional role in the cytoarchitecture of neurons and GABAergic synaptic connectivity. *J Biol Chem* **2009,** 284, (25), 17253-17265.

28. Takano, K.; Kojima, T.; Sawada, N.; Himi, T., Role of tight junctions in signal transduction: an update. *Excli j* **2014,** 13, 1145-62.

29. Huo, L.; Wen, W.; Wang, R.; Kam, C.; Xia, J.; Feng, W.; Zhang, M., Cdc42-dependent formation of the ZO-1/MRCKβ complex at the leading edge controls cell migration. *Embo j* **2011,** 30, (4), 665-78.

30. Hwangbo, C.; Tae, N.; Lee, S.; Kim, O.; Park, O. K.; Kim, J.; Kwon, S. H.; Lee, J. H., Syntenin regulates TGF-β1-induced Smad activation and the epithelial-to-mesenchymal transition by inhibiting caveolin-mediated TGF-β type I receptor internalization. *Oncogene* **2016,** 35, (3), 389-401.

31. Ding, Y.; Caberoy, N. B.; Guo, F.; LeBlanc, M. E.; Zhang, C.; Wang, W.; Wang, F.; Chen, R.; Li, W., Reticulocalbin-1 facilitates microglial phagocytosis. *PLoS One* **2015,** 10, (5), e0126993.

32. Sangwan, V.; Paliouras, G. N.; Abella, J. V.; Dubé, N.; Monast, A.; Tremblay, M. L.; Park, M., Regulation of the Met receptor-tyrosine kinase by the protein-tyrosine phosphatase 1B and T-cell phosphatase. *J Biol Chem* **2008,** 283, (49), 34374-83.

33. Hanna, R. A.; Campbell, R. L.; Davies, P. L., Calcium-bound structure of calpain and its mechanism of inhibition by calpastatin. *Nature* **2008,** 456, (7220), 409-412.

34. Lebart, M.-C.; Benyamin, Y., Calpain involvement in the remodeling of cytoskeletal anchorage complexes. *The FEBS Journal* **2006,** 273, (15), 3415-3426.

35. Kobayashi, T.; Miyashita, T.; Murayama, T.; Toyoshima, Y. Y., Dynactin has two antagonistic regulatory domains and exerts opposing effects on dynein motility. *PLoS One* **2017,** 12, (8), e0183672.

36. Kopecki, Z.; O'Neill, G. M.; Arkell, R. M.; Cowin, A. J., Regulation of focal adhesions by flightless i involves inhibition of paxillin phosphorylation via a Rac1-dependent pathway. *J Invest Dermatol* **2011,** 131, (7), 1450-9.

37. Yu, H.; Shen, H.; Zhang, Y.; Zhong, F.; Liu, Y.; Qin, L.; Yang, P., CAV1 promotes HCC cell progression and metastasis through Wnt/β-catenin pathway. *PLoS One* **2014,** 9, (9), e106451.

38. Fan, J.; Fong, T.; Chen, X.; Chen, C.; Luo, P.; Xie, H., Glia maturation factor-β: a potential therapeutic target in neurodegeneration and neuroinflammation. *Neuropsychiatr Dis Treat* **2018,** 14, 495-504.

39. Ott, E. B.; van den Akker, N. M. S.; Sakalis, P. A.; Gittenberger-de Groot, A. C.; Te Velthuis, A. J. W.; Bagowski, C. P., The lim domain only protein 7 is important in zebrafish heart development. *Developmental Dynamics* **2008,** 237, (12), 3940-3952.

40. Shin, J. H.; Kim, Y. N.; Kim, I. Y.; Choi, D. H.; Yi, S. S.; Seong, J. K., Increased Cell Proliferations and Neurogenesis in the Hippocampal Dentate Gyrus of Ahnak Deficient Mice. *Neurochem Res* **2015,** 40, (7), 1457-62.

41. Ma, L.; Greenwood, J. A.; Schachner, M., CRP1, a protein localized in filopodia of growth cones, is involved in dendritic growth. *J Neurosci* **2011,** 31, (46), 16781-91.

42. Duan, H. O.; Simpson-Haidaris, P. J., Functional analysis of interleukin 6 response elements (IL-6REs) on the human gamma-fibrinogen promoter: binding of hepatic Stat3 correlates negatively with transactivation potential of type II IL-6REs. *J Biol Chem* **2003,** 278, (42), 41270-81.

43. Crende, O.; Sabatino, M.; Valcárcel, M.; Carrascal, T.; Riestra, P.; López-Guerrero, J. A.; Nagore, E.; Mandruzzato, S.; Wang, E.; Marincola, F. M.; Vidal-Vanaclocha, F., Metastatic lesions with and without interleukin-18-dependent genes in advanced-stage melanoma patients. *Am J Pathol* **2013,** 183, (1), 69-82.

44. Klein, W. M.; Wu, B. P.; Zhao, S.; Wu, H.; Klein-Szanto, A. J.; Tahan, S. R., Increased expression of stem cell markers in malignant melanoma. *Mod Pathol* **2007,** 20, (1), 102-7.

45. Flørenes, V. A.; Holm, R.; Myklebost, O.; Lendahl, U.; Fodstad, O., Expression of the neuroectodermal intermediate filament nestin in human melanomas. *Cancer Res* **1994,** 54, (2), 354-6.

46. Neradil, J.; Veselska, R., Nestin as a marker of cancer stem cells. *Cancer Sci* **2015,** 106, (7), 803-11.

47. Michalczyk, K.; Ziman, M., Nestin structure and predicted function in cellular cytoskeletal organisation. *Histol Histopathol* **2005,** 20, (2), 665-71.

48. Liu, C. Y.; Lin, H. H.; Tang, M. J.; Wang, Y. K., Vimentin contributes to epithelial-mesenchymal transition cancer cell mechanics by mediating cytoskeletal organization and focal adhesion maturation. *Oncotarget* **2015,** 6, (18), 15966-83.

49. Kosnopfel, C.; Sinnberg, T.; Sauer, B.; Busch, C.; Niessner, H.; Schmitt, A.; Forchhammer, S.; Grimmel, C.; Mertens, P. R.; Hailfinger, S.; Dunn, S. E.; Garbe, C.; Schittek, B., YB-1 Expression and Phosphorylation Regulate Tumorigenicity and Invasiveness in Melanoma by Influencing EMT. *Mol Cancer Res* **2018,** 16, (7), 1149-1160.

50. Sinnberg, T.; Sauer, B.; Holm, P.; Spangler, B.; Kuphal, S.; Bosserhoff, A.; Schittek, B., MAPK and PI3K/AKT mediated YB‐1 activation promotes melanoma cell proliferation which is counteracted by an autoregulatory loop. *Experimental dermatology* **2012,** 21, (4), 265-270.

51. Guo, D.; Lui, G. Y. L.; Lai, S. L.; Wilmott, J. S.; Tikoo, S.; Jackett, L. A.; Quek, C.; Brown, D. L.; Sharp, D. M.; Kwan, R. Y. Q.; Chacon, D.; Wong, J. H.; Beck, D.; van Geldermalsen, M.; Holst, J.; Thompson, J. F.; Mann, G. J.; Scolyer, R. A.; Stow, J. L.; Weninger, W.; Haass, N. K.; Beaumont, K. A., RAB27A promotes melanoma cell invasion and metastasis via regulation of pro-invasive exosomes. *International Journal of Cancer* **2019,** 144, (12), 3070-3085.

52. Rocco, M.; Malorni, L.; Cozzolino, R.; Palmieri, G.; Rozzo, C.; Manca, A.; Parente, A.; Chambery, A., Proteomic Profiling of Human Melanoma Metastatic Cell Line Secretomes. *Journal of Proteome Research* **2011,** 10, (10), 4703-4714.

53. Koman, I. E.; Commane, M.; Paszkiewicz, G.; Hoonjan, B.; Pal, S.; Safina, A.; Toshkov, I.; Purmal, A. A.; Wang, D.; Liu, S.; Morrison, C.; Gudkov, A. V.; Gurova, K. V., Targeting FACT complex suppresses mammary tumorigenesis in Her2/neu transgenic mice. *Cancer Prev Res (Phila)* **2012,** 5, (8), 1025-35.

54. Fleyshman, D.; Prendergast, L.; Safina, A.; Paszkiewicz, G.; Commane, M.; Morgan, K.; Attwood, K.; Gurova, K., Level of FACT defines the transcriptional landscape and aggressive phenotype of breast cancer cells. *Oncotarget* **2017,** 8, (13), 20525-20542.

55. Strezoska, Z.; Pestov, D. G.; Lau, L. F., Functional inactivation of the mouse nucleolar protein Bop1 inhibits multiple steps in pre-rRNA processing and blocks cell cycle progression. *J Biol Chem* **2002,** 277, (33), 29617-25.

56. Rohrmoser, M.; Hölzel, M.; Grimm, T.; Malamoussi, A.; Harasim, T.; Orban, M.; Pfisterer, I.; Gruber-Eber, A.; Kremmer, E.; Eick, D., Interdependence of Pes1, Bop1, and WDR12 controls nucleolar localization and assembly of the PeBoW complex required for maturation of the 60S ribosomal subunit. *Mol Cell Biol* **2007,** 27, (10), 3682-94.

57. Gupta, R.; Bugide, S.; Wang, B.; Green, M. R.; Johnson, D. B.; Wajapeyee, N., Loss of BOP1 confers resistance to BRAF kinase inhibitors in melanoma by activating MAP kinase pathway. *Proceedings of the National Academy of Sciences* **2019,** 116, (10), 4583-4591.

58. Jia, F.; Wu, Q.; Wang, Z.; Zhang, M.; Yuan, S.; Che, Y.; Li, B.; Hu, Z.; Hu, X., BOP1 Knockdown Attenuates Neointimal Hyperplasia by Activating p53 and Inhibiting Nascent Protein Synthesis. *Oxid Med Cell Longev* **2021,** 2021, 5986260.

59. Li, W.; Song, P.; Zhao, M.; Gao, L.; Xie, J.; You, C., BOP1 Used as a Novel Prognostic Marker and Correlated with Tumor Microenvironment in Pan-Cancer. *J Oncol* **2021,** 2021, 3603030.

60. Quidville, V.; Alsafadi, S.; Goubar, A.; Commo, F.; Scott, V.; Pioche-Durieu, C.; Girault, I.; Baconnais, S.; Le Cam, E.; Lazar, V.; Delaloge, S.; Saghatchian, M.; Pautier, P.; Morice, P.; Dessen, P.; Vagner, S.; André, F., Targeting the deregulated spliceosome core machinery in cancer cells triggers mTOR blockade and autophagy. *Cancer Res* **2013,** 73, (7), 2247-58.

61. Mi, L.; Qi, Q.; Ran, H.; Chen, L.; Li, D.; Xiao, D.; Wu, J.; Cai, Y.; Zhang, S.; Li, Y.; Li, B.; Xie, J.; Huang, H.; Li, T.; Zhou, T.; Li, A.; Qi, J.; Li, F.; Man, J., Suppression of Ribosome Biogenesis by Targeting WD Repeat Domain 12 (WDR12) Inhibits Glioma Stem-Like Cell Growth. *Front Oncol* **2021,** 11, 751792.

62. Yildiz, L.; Kefeli, M.; Aydin, O.; Kandemir, B., Fascin expression in melanocytic lesions of the skin. *Eur J Dermatol* **2009,** 19, (5), 445-50.

63. Ma, Y.; Faller, W. J.; Sansom, O. J.; Brown, E. R.; Doig, T. N.; Melton, D. W.; Machesky, L. M., Fascin expression is increased in metastatic lesions but does not correlate with progression nor outcome in melanoma. *Melanoma Res* **2015,** 25, (2), 169-72.

64. Bosco, B.; Rossi, A.; Rizzotto, D.; Hamadou, M. H.; Bisio, A.; Giorgetta, S.; Perzolli, A.; Bonollo, F.; Gaucherot, A.; Catez, F.; Diaz, J. J.; Dassi, E.; Inga, A., DHX30 Coordinates Cytoplasmic Translation and Mitochondrial Function Contributing to Cancer Cell Survival. *Cancers (Basel)* **2021,** 13, (17).

65. Li, S.; Ma, J.; Zheng, A.; Song, X.; Chen, S.; Jin, F., DEAD-box helicase 27 enhances stem cell-like properties with poor prognosis in breast cancer. *Journal of Translational Medicine* **2021,** 19, (1), 334.

66. Paulitschke, V.; Haudek-Prinz, V.; Griss, J.; Berger, W.; Mohr, T.; Pehamberger, H.; Kunstfeld, R.; Gerner, C., Functional Classification of Cellular Proteome Profiles Support the Identification of Drug Resistance Signatures in Melanoma Cells. *Journal of Proteome Research* **2013,** 12, (7), 3264-3276.

67. Smith, C.; Dolat, L.; Angelis, D.; Forgacs, E.; Spiliotis, E. T.; Galkin, V. E., Septin 9 Exhibits Polymorphic Binding to F-Actin and Inhibits Myosin and Cofilin Activity. *Journal of Molecular Biology* **2015,** 427, (20), 3273-3284.

68. Gonzales, M.; Weksler, B.; Tsuruta, D.; Goldman, R. D.; Yoon, K. J.; Hopkinson, S. B.; Flitney, F. W.; Jones, J. C., Structure and function of a vimentin-associated matrix adhesion in endothelial cells. *Molecular Biology of the Cell* **2001,** 12, (1), 85-100.

69. Li, M.; Zhang, B.; Sun, B.; Wang, X.; Ban, X.; Sun, T.; Liu, Z.; Zhao, X., A novel function for vimentin: the potential biomarker for predicting melanoma hematogenous metastasis. *J Exp Clin Cancer Res* **2010,** 29, (1), 109.

70. Garcia, H.; Miecznikowski, J. C.; Safina, A.; Commane, M.; Ruusulehto, A.; Kilpinen, S.; Leach, R. W.; Attwood, K.; Li, Y.; Degan, S.; Omilian, A. R.; Guryanova, O.; Papantonopoulou, O.; Wang, J.; Buck, M.; Liu, S.; Morrison, C.; Gurova, K. V., Facilitates chromatin transcription complex is an "accelerator" of tumor transformation and potential marker and target of aggressive cancers. *Cell Rep* **2013,** 4, (1), 159-73.

71. Gurova, K. V.; Garcia, H.; Miecznikowski, J.; Omilian, A. R.; Morrison, C., Level of SSRP1 in cancer as a prognostic marker of aggressive disease. *American Journal of Clinical Pathology* **2013,** 140, (suppl_1), A152-A152.
